# TNF superfamily member 14 drives post-influenza depletion of alveolar macrophages, enabling secondary pneumococcal pneumonia

**DOI:** 10.1172/JCI185390

**Published:** 2025-11-18

**Authors:** Christina Malainou, Christin Peteranderl, Maximiliano Ruben Ferrero, Ana Ivonne Vazquez-Armendariz, Ioannis Alexopoulos, Katharina Franz, Klara Knippenberg, Julian Better, Mohammad Estiri, Cheng-Yu Wu, Hendrik Schultheis, Judith Bushe, Maria-Luisa del Rio, Jose Ignacio Rodriguez-Barbosa, Klaus Pfeffer, Stefan Günther, Mario Looso, Achim Dieter Gruber, István Vadász, Ulrich Matt, Susanne Herold

**Affiliations:** 1 Department of Medicine V, Internal Medicine, Infectious Diseases and Infection Control, Universities of Giessen and Marburg Lung Center (UGMLC), member of the German Center for Lung Research (DZL), member of the German Center for Infection Research (DZIF), Justus Liebig University Giessen, Giessen, Germany.; 2 Institute of Lung Health (ILH), Justus Liebig University Giessen, Giessen, Germany.; 3 Excellence Cluster Cardio-Pulmonary Institute (CPI), Hessen, Germany.; 4 Biomedicine Research Institute of Buenos Aires – CONICET – Partner Institute of the Max Planck Society (IBioBA-MPSP), Buenos Aires, Argentina.; 5 Max Planck Institute for Heart and Lung Research, Bad Nauheim, Germany.; 6 University of Bonn, Transdisciplinary Research Area Life and Health, Organoid Biology, Life and Medical Sciences Institute, Bonn, Germany.; 7 Department of Internal Medicine II, Universities of Giessen and Marburg Lung Center, University Hospital Giessen, Justus Liebig University, Member of the German Center for Lung Research (DZL), Giessen, Germany.; 8 Department of Veterinary Pathology, Free University Berlin, Berlin, Germany.; 9 Transplantation Immunobiology Section, Institute of Molecular Biology, Genomics and Proteomics (INBIOMIC), University of León, León, Spain.; 10 Institute of Medical Microbiology and Hospital Hygiene, Heinrich Heine University Düsseldorf, Düsseldorf, Germany.

**Keywords:** Cell biology, Infectious disease, Bacterial infections, Influenza

## Abstract

Secondary bacterial infection, often caused by *Streptococcus pneumoniae*, is one of the most frequent and severe complications of influenza A virus–induced (IAV-induced) pneumonia. Phenotyping of the pulmonary immune cell landscape after IAV infection revealed a substantial depletion of the tissue-resident alveolar macrophage (TR-AM) population at day 7, which was associated with increased susceptibility to *S. pneumoniae* outgrowth. To elucidate the molecular mechanisms underlying TR-AM depletion, and to define putative targets for treatment, we combined single-cell transcriptomics and cell-specific PCR profiling in an unbiased manner, using in vivo models of IAV infection and IAV and *S. pneumoniae* coinfection. The TNF superfamily 14 (TNFSF14) ligand/receptor axis was revealed as the driving force behind post-influenza TR-AM death during the early infection phase, enabling the transition to pneumococcal pneumonia, whereas intrapulmonary transfer of genetically modified TR-AMs and antibody-mediated neutralization of specific pathway components alleviated disease severity. With mainly neutrophilic expression and high abundance in the bronchoalveolar fluid of patients with severe virus-induced acute respiratory distress syndrome, TNFSF14 emerged as a key determinant of virus-driven lung injury. Targeting the TNFSF14-mediated intercellular communication network in the virus-infected lung can, therefore, improve host defense, minimizing the risk of subsequent bacterial pneumonia and ameliorating the disease outcome.

## Abstract 2

Targeting neutrophil-driven alveolar macrophage death in severe influenza pneumonia strengthens host defense and prevents the transition to secondary bacterial infection, improving patient outcome.

## Introduction

Bacterial pneumonia, often caused by *Streptococcus pneumoniae*, is one of the most common complications of primary influenza A virus (IAV) infection, increasing the risk of death, intensive care unit (ICU) admission, and requirement for mechanical ventilation (1). While strengthening the host defense offers a potential alternative to antibiotics amid globally rising resistance, progress is limited by a poor understanding of the immune mechanisms behind severe influenza and the transition to post–viral bacterial pneumonia. Among these, virus-induced depletion of the tissue-resident alveolar macrophage (TR-AM) pool is considered a key factor in promoting secondary bacterial pneumonia, alongside epithelial damage, influx of proinflammatory cells, and impaired mechanical clearance (2, 3). TR-AM numbers remain relatively unchanged during homeostasis, with the main function of the cells being surfactant clearance and containment of minor infections (4). This tolerogenic programming, however, can be overridden by abundant viral presence, leading to a proinflammatory phenotypic switch, including extensive cytokine release and phagocytosis of viral particles and apoptotic cells (4, 5). TR-AM loss, often observed after severe infection and notoriously known as the “TR-AM disappearance reaction” (6, 7), dramatically increases IAV-associated mortality (8, 9), however, the specific pathomechanisms behind it remain elusive.

The TNF superfamily (TNFSF) involves a variety of structurally homologous ligands with multiple functions during development, homeostasis, and tissue response to injury (10). TNFSF14 or LIGHT (homologous to lymphotoxin, exhibits inducible expression, and competes with herpes simplex virus [HSV] glycoprotein D for the herpes virus entry mediator [HVEM], a receptor expressed by T lymphocytes) is widely expressed on cells of the hematopoietic compartment (11, 12). In the lung, TNFSF14 has been associated with airway remodeling in asthma, idiopathic pulmonary fibrosis, systemic sclerosis models (13, 14), and, more recently, disease severity in COVID-19 (15, 16). Still, little is known regarding the pathomechanistic role of TNFSF14 in virus-induced pneumonia.

TNFSF14 binds to 3 different receptors: type I transmembrane lymphotoxin β receptor (LTβR), HVEM, also known as TNF receptor superfamily 14 (TNFRSF14), and decoy receptor 3 (DcR3), which is only found in primates (10). TNFSF14 receptors have a broad distribution on immune, stromal, and parenchymal cells (17) and orchestrate distinct intracellular pathways. The outcome of TNFSF14 crosslinking to its receptors, therefore, heavily relies on disease context and microenvironmental cues (10). Here, we sought to investigate the molecular mechanisms of post-influenza TR-AM loss and its consequences for host defense in a model of IAV and IAV/*S. pneumoniae* infection, with the aim of identifying any putative targets for immune-based pneumonia treatment options.

## Results

### Severe IAV infection increases susceptibility to secondary pneumococcal infection.

**Figure 1:**
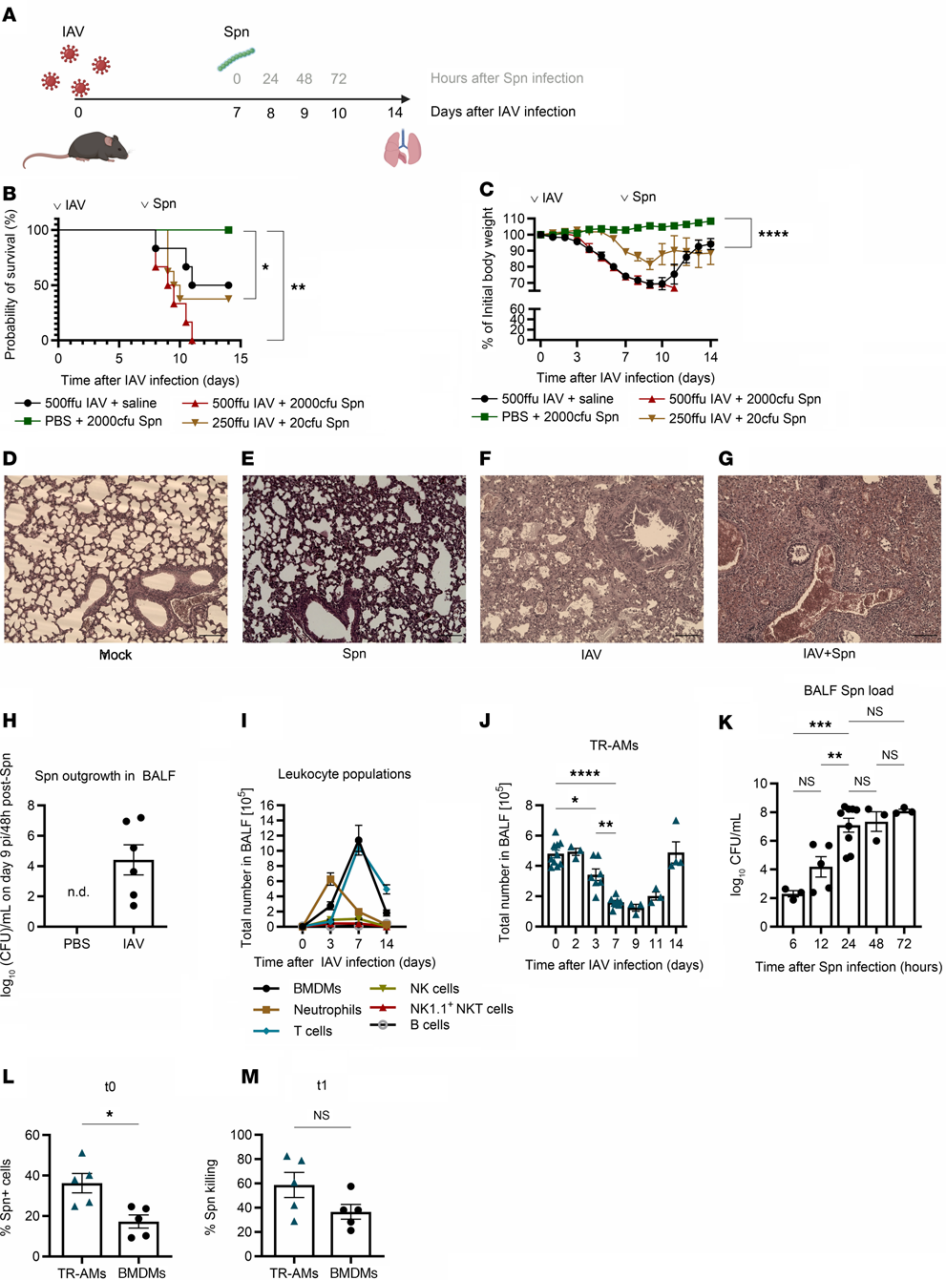
IAV infection increases susceptibility to secondary pneumococcal infection. (**A**) Schematic representation of the coinfection model, with pneumococcal infection taking place 7 days after IAV infection. Figure created with BioRender.com. (**B** and **C**) Survival (**B**) and weight loss (**C**) after coinfection of WT mice with IAV and *S. pneumoniae* (*n* = 6–8 mice; data were pooled from 5 independent experiments). (**D**–**G**) Representative histological images of mock-infected mice (**D**), *S. pneumoniae*–infected mice (**E**), IAV-infected mice (**F**), or mice infected with IAV 7 days prior to *S. pneumoniae* infection (**G**). Lungs were harvested 10 days after IAV infection. Scale bars: 100 μm. Data were pooled from 2 independent experiments. (**H**) Bacterial load in the BALF of IAV- or mock-infected mice 9 days after IAV infection and 48 hours after *S. pneumoniae* infection (mean ± SEM, *n* = 6–9; data are representative of 3 independent experiments). (**I**) Leukocyte populations including neutrophils (*n* = 7–9), BMDMs (*n* = 8–9), NK cells (*n* = 7–9), T cells (*n* = 3–9), NK1.1^+^ NKT cells (*n* = 3–9), and B cells (*n* = 5–9) in the BALF of IAV-infected mice 0–14 days p.i. (mean ± SEM; data were pooled from 16 independent experiments). (**J**) TR-AM population during the IAV infection course (mean ± SEM, *n* = 3–10; data were pooled from 6 independent experiments). (**K**) BALF bacterial load 6–72 hours after pneumococcal superinfection performed 7 days after IAV infection (mean ± SEM, *n* = 3–9 per time point; data were pooled from 3 independent experiments). (**L** and **M**) *S. pneumoniae* phagocytosis capacity (**L**) depicted as the percentage of *S. pneumoniae*^+^ cells 10 minutes (*t0*) after infection and killing capacity (**M**) (percent killing at *t1* over *t0*) for day 8 TR-AMs and BMDMs (*n* = 5; data are representative of 3 independent experiments). *P < 0.05, **P < 0.01, and ****P < 0.0001, by log-rank (Mantel-Cox) test (**B**), unpaired, 2-tailed Student’s *t* test (**L** and **M**), and 1-way ANOVA with Tukey’s post hoc test (**J** and **K**). Spn, *S. pneumoniae*.

To elucidate the pathomechanisms behind TR-AM death after severe IAV infection and its effect on the establishment of secondary bacterial pneumonia, we established a robust coinfection model (Figure 1A). Disease severity after viral, bacterial, or coinfection was shown to vary in a pathogen- and infection dose–dependent manner. Orotracheal (o.t.) infection of C57BL/6 WT mice with 500 foci-forming units (FFU) IAV A/PR/8/34 decreased mouse survival by 50% 14 days after infection, whereas intranasal (i.n.) infection with 2,000 CFU *S. pneumoniae* serotype 3 (PN36 NCTC7978) did not affect survival (Figure 1B) or weight loss (Figure 1C). However, IAV infection 7 days prior to pneumococcal infection caused massive leukocyte infiltration (Figure 1, D–G) and a 100% lethal outcome (Figure 1B). Upon use of lower IAV and *S. pneumoniae* doses (250 FFU/20 CFU on day 7 after IAV infection), the average survival percentage was 37.5% (Figure 1B). Despite the low infection doses, bacterial load in the bronchoalveolar lavage fluid (BALF) of previously IAV-infected mice was remarkably high 48 hours after pneumococcal infection, whereas PBS-pretreated mice completely cleared the infection (Figure 1H), suggesting an IAV-associated impaired immune response against invading *S. pneumoniae*. We therefore characterized the leukocyte landscape of the IAV-infected lung, as distinct immune cell populations and their interactions can differentially affect post-influenza bacterial clearance (3, 18, 19). Flow cytometric profiling of BALF leukocytes (gating strategy depicted in Supplemental Figure 1A; supplemental material available online with this article; https://doi.org/10.1172/JCI185390DS1) revealed cell-specific kinetics over the infection course (Figure 1I). Of note, BALF TR-AM numbers, which started significantly declining on post-infection (p.i.) day 3, were almost completely depleted between p.i. days 7–11 (Figure 1J). We also observed similar results for lung-tissue leukocytes (gating strategy shown in Supplemental Figure 1B), which could not be acquired through BAL because of their sessile nature (Supplemental Figure 2A) (20) or extra-alveolar location (Supplemental Figure 2, B–M). Upon coinfection, BALF bacterial outgrowth was observed 24 hours after pneumococcal infection (Figure 1K), coinciding with the period of maximum TR-AM depletion, despite the abundant presence of bone marrow–derived macrophages (BMDMs). TR-AMs presented a greater *S. pneumoniae* phagocytosis capacity (Figure 1L) and no inferiority in killing capacity to the infection-driven proinflammatory BMDMs (Figure 1M), highlighting the importance of TR-AM preservation for maintaining the intact host defense. To address this, we sought to identify the molecular underpinnings of post-influenza TR-AM death.

### Post-influenza TR-AM death involves the activation of caspase 8.

**Figure 2:**
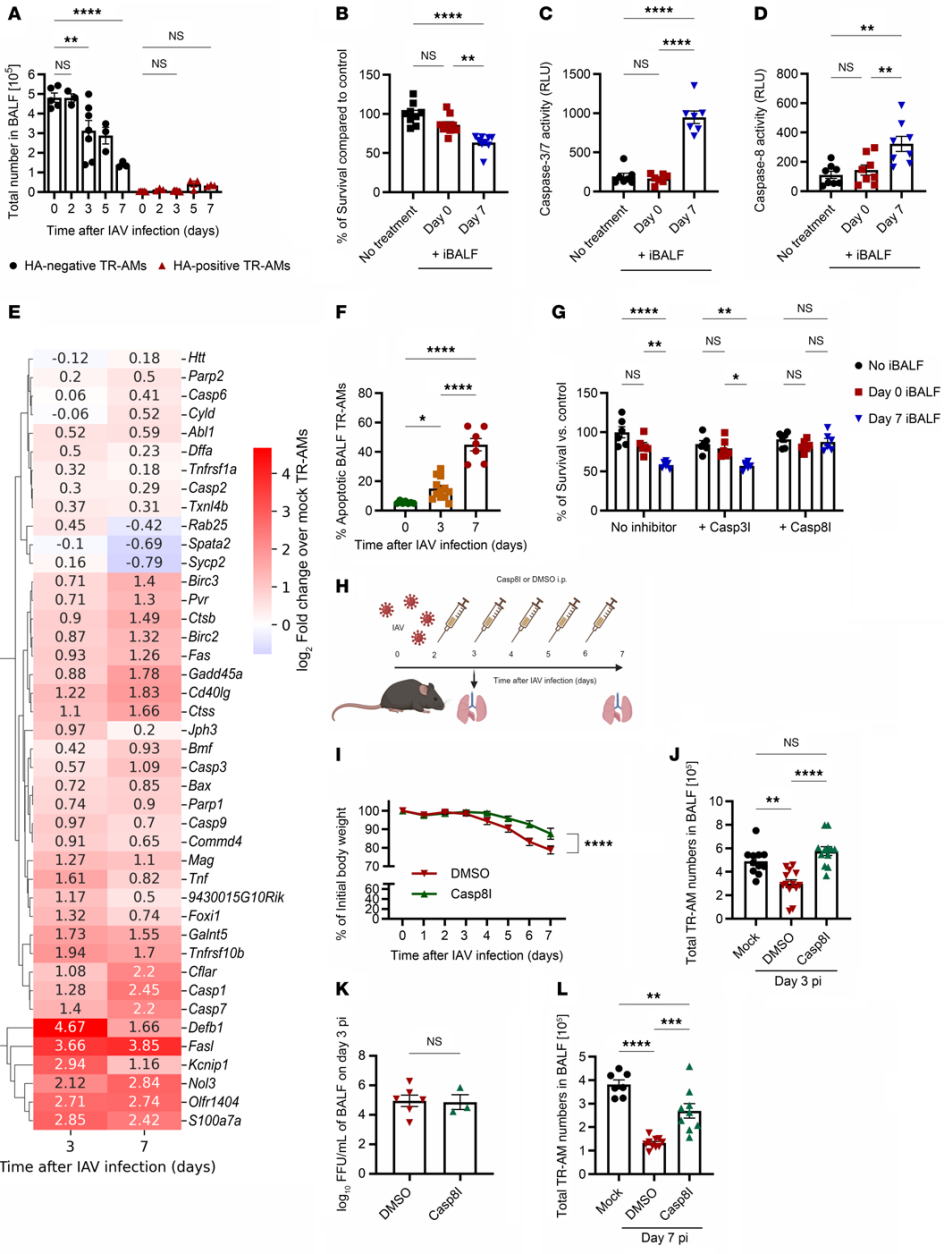
Caspase 8 is involved in virus-independent, post-influenza TR-AM death. (**A**) Quantification of viral HA^–^ and HA^+^ TR-AMs after IAV infection (*n* = 3–7; data indicate the mean ± SEM and were pooled from 3 independent experiments). (**B**) TR-AM survival following 24 hours of treatment with iBALF (*n* = 9 per group; data represent the mean ± SEM and were pooled from 3 independent experiments). (**C** and **D**) Caspase 3/-7 (**C**) and caspase 8 activity (**D**) after iBALF TR-AM treatment (*n* = 7–8; data represent the mean ± SEM and were pooled from 3 independent experiments). (**E**) Heatmap depicting the average fold changes of cell death–related genes in flow-sorted, HA^–^, mock, day 3, and day 7 p.i. BALF TR-AMs (*n* = 3–7 per time point; data were pooled from 4 independent experiments). Significance was determined by 2-tailed Student’s *t* test for the log_2_ fold-change values of each gene in the compared groups. (**F**) Percentage of apoptotic TR-AMs on days 0, 3, and 7 p.i. (*n* = 7–12; data represent the mean ± SEM and were pooled from 4 independent experiments). (**G**) Colorimetric viability assay following naive TR-AM treatment with iBALF after 3 hours of pretreatment with 50 μM of a specific caspase inhibitor (*n* = 6; data represent the mean ± SEM and were pooled from 2 independent experiments). (**H**) Experimental layout for caspase 8 inhibition in vivo experiments. Figure created with BioRender.com. (**I**) Weight loss after IAV infection and caspase 8 inhibition (*n* = 8–11; data were pooled from 6 independent experiments). (**J**) BALF TR-AMs on day 3 after in vivo caspase 8 inhibition (*n* = 11–13; data represent the mean ± SEM and were pooled from 4 independent experiments). (**K**) BALF viral titers on day 3 after in vivo caspase 8 inhibition (*n* = 3–6; data represent the mean ± SEM and were pooled from 2 independent experiments). (**L**) BALF TR-AMs on day 7 after in vivo caspase 8 inhibition (*n* = 7–10; data represent the mean ± SEM and were pooled from 7 independent experiments). *P < 0.05, **P < 0.01, ***P < 0.001, and ****P < 0.0001, by 2-tailed Student’s *t* test (**I** [AUC for the compared groups] and **K**), 1-way ANOVA with Tukey’s post hoc test (**B**–**D**, **F**, **J** and **L**), and 2-way ANOVA with Tukey’s post hoc test (**A** and **G**).

Direct viral infection can lead to epithelial cell apoptosis in IAV-induced pneumonia (21, 22), raising the question of whether this also drives post-IAV TR-AM depletion. Flow cytometric analysis revealed only a small number of IAV-infected TR-AMs (quantified by viral HA expression) with no significant increase over the course of the infection (Figure 2A). The majority of HA^–^ cells had been depleted by day 7 p.i. (Figure 2A), implying the involvement of a different mechanism with a much stronger effect. In accordance with that, when naive TR-AMs were treated ex vivo with virus- and cell-free BALF from IAV-infected mice (iBALF) from day 7 p.i. (day of maximum depletion), we observed a significant decrease in TR-AM survival (Figure 2B) as well as an increase in caspase 3/-7 (Figure 2C) and caspase 8 activity (Figure 2D). Transcriptome analysis of flow-sorted HA^–^ TR-AMs on days 3 and 7 p.i. based on a cell death gene array revealed an upregulation of multiple apoptosis-related genes, such as *Bax*, *Cd40lg*, and *Cflar*, and necrosis-related genes, including *Bmf*, *Commd4*, *Defb1*, and *Parp1* (Figure 2E and Supplemental Data 2, cell death arrays WT data). The fold changes of upregulated genes did not differ significantly from baseline, as cell death is mainly regulated on a (post)translational level (23). Concomitant flow cytometric analysis, however, revealed a remarkable increase in apoptotic TR-AMs over the infection course (Figure 2F and gating strategy in Supplemental Figure 3A). As a result, we posed the question of whether apoptosis inhibition would improve TR-AM survival. Following preincubation with a nontoxic concentration (Supplemental Figure 3, B and C) of 50 μM of a specific caspase 3 (Z-DEVD-FMK) or caspase 8 (Z-IETD-FMK) inhibitor, we treated naive TR-AMs with iBALF. Whereas caspase 3 inhibition only showed a negligibly protective effect, TR-AM death was completely abrogated in the caspase 8 inhibition group (Figure 2G). When mice were treated with daily s.c. injections of the caspase 8 inhibitor (see schematic of the experimental layout in Figure 2H), we observed an attenuated weight loss up to day 7 p.i. (Figure 2I). Caspase 8 inhibition fully protected the TR-AM pool on day 3 p.i. (Figure 2J), without affecting viral titers (Figure 2K), and significantly mitigated TR-AM loss on day 7 p.i. (Figure 2L). Caspase 8 is a known orchestrator of cell death that is typically activated upon the crosslinking of a soluble ligand to a death receptor (24), which, together with the primarily virus-independent TR-AM apoptosis, hinted at a soluble ligand as a driver of TR-AM death.

### IAV pneumonia sensitizes TR-AMs to TNFSF14 ligation.

**Figure 3:**
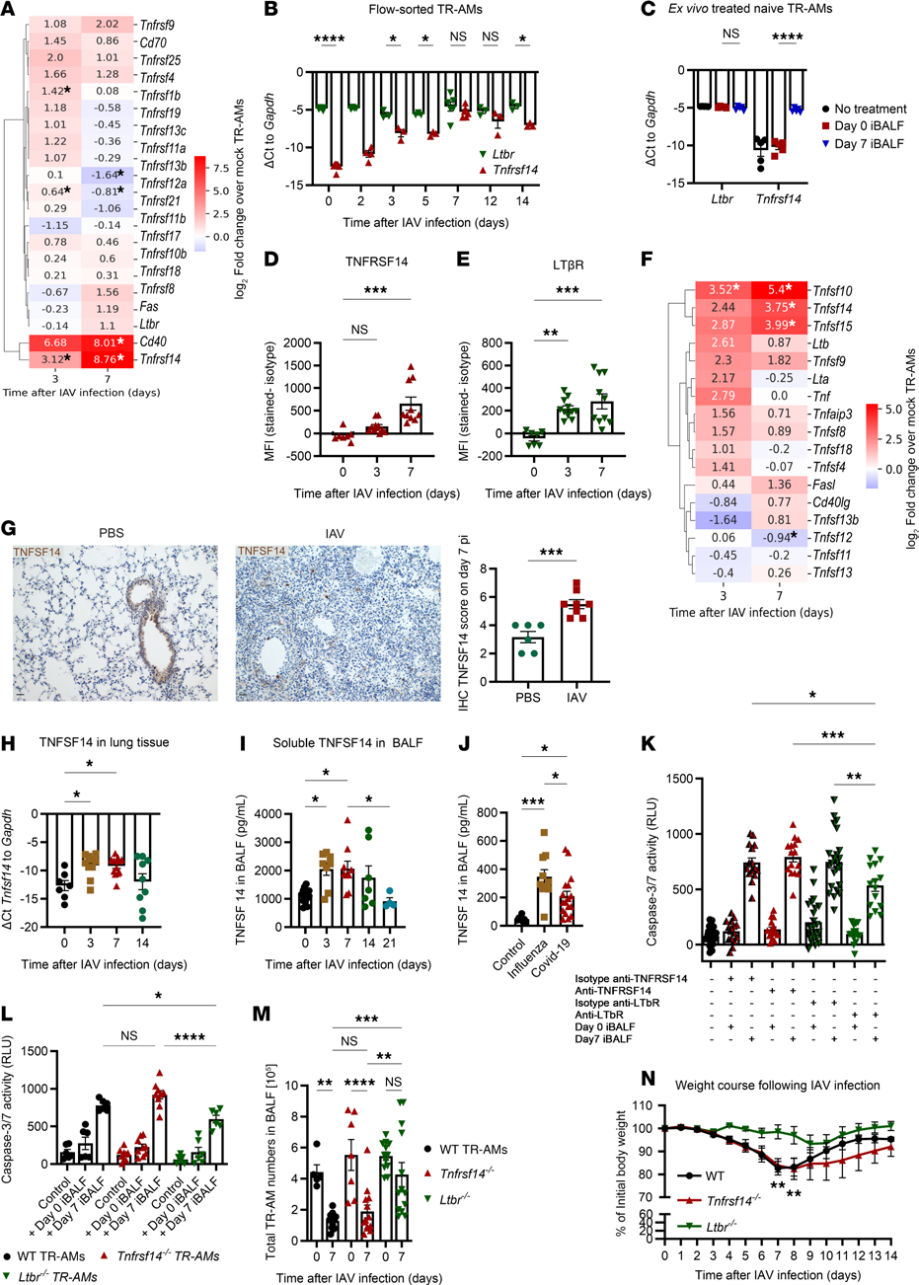
IAV infection leads to increased expression of the TNFSF14 ligand/receptor axis. (**A**) Fold change of TNFSF receptor genes in mock-infected, day 3–infected, and day 7–infected TR-AMs (*n* = 5–6; data were pooled from 3 experiments). (**B**) TR-AM *Tnfrsf14* and *Ltbr* gene expression over the course of the IAV infection (*n* = 3–6; data were pooled from 2 experiments). (**C**) TR-AM *Tnfrsf14* and *Ltbr* gene expression after ex vivo iBALF treatment (*n* = 5; data are representative of 3 experiments). (**D** and **E**) TR-AM TNFRSF14 (**D**) and LTβR (**E**) expression (*n* = 7–11; data were pooled from 6 experiments). (**F**) Fold change of TNFSF ligand genes in mock-infected, day 3–infected, and day 7–infected TR-AMs (*n* = 5–6, data were pooled from 3 independent experiments). (**G**) IHC analysis for TNFSF14 expression after mock (PBS) or IAV infection. Scale bar: 25 μm (*n* = 6–8; data were pooled from 2 experiments). (**H**) *Tnfsf14* gene expression in the lungs of IAV-infected animals (*n* = 7–8; data were pooled from 3 experiments). (**I**) BALF TNFSF14 measured by ELISA (*n* = 4–15; data were pooled from 7 experiments). (**J**) Soluble TNFSF14 in the BALF of patients with severe viral pneumonia (*n* = 8–17 per group; data were pooled from 3 experiments). (**K**) Caspase 3/-7 activity after iBALF treatment of naive WT TR-AMs, following anti-LTβR or anti-TNFRSF14 blocking (*n* = 15–28; data were pooled from 6 experiments). (**L**) Caspase 3/-7 activity after iBALF treatment of naive WT, *Tnfrsf14^–/–^*, and *Ltbr^–/–^* TR-AMs (*n* = 6–9; data were pooled from 4 experiments). (**M**) TR-AM numbers for WT, *Tnfrsf14^–/–^*, and *Ltbr^–/–^* mice on days 0 and 7 p.i. (*n* = 5–13; data were pooled from 12 experiments, WT controls including values are depicted in Figure 1J). (**N**) Body weight of WT, *Tnfsf14^–/–^*, and *Ltbr^–/–^* mice over the course of IAV infection (*n* = 5 per group; data were pooled from 3 experiments and represent the mean ± SEM). *P < 0.05, **P < 0.01, ***P < 0.001, and ****P < 0.0001, by unpaired, 2-tailed Student’s *t* test (**G**), 1-way, with Tukey’s post hoc test (**D**, **E**, **H**, and **K**), 2-way ANOVA with Tukey’s post hoc test (**B**, **C**, **L**–**N** [comparison of weight loss among the 3 groups at each time point after infection]), and Kruskal-Wallis test followed by Dunn′s post hoc comparison test (**I**). For the heatmaps in **A** and **F**, a 2-tailed Student’s *t* test was performed for the log_2_ fold-change values of each gene in the compared groups.

Death-inducing members of the TNFSF have been associated with promoting alveolar epithelial cell (AEC) death and driving post-IAV lung injury (18, 25, 26). As such, we hypothesized that a TNFSF member could be involved in post-IAV TR-AM death and analyzed gene expression patterns of receptors and ligands belonging to the TNFSF signaling network in flow-sorted HA^–^ TR-AMs from mock-infected and infected mice on days 3 and 7 p.i. TNFRSF14, a receptor for TNFSF14, showed marked upregulation at both time points (Figure 3A and Supplemental Data 1, TNF signaling arrays WT data). TNFRSF14 demonstrated a significant increase in mRNA and cell-surface protein expression levels over the infection course and after ex vivo TR-AM stimulation with iBALF (Figure 3, B–D). LTβR, the competitive receptor for TNFSF14, presented no changes in transcriptional regulation, yet a distinct increase in protein expression (Figure 3, B, C, and E). On day 3 p.i., the majority of TR-AMs stained positive for TNFSF14 receptors (Supplemental Figure 4A). With regard to TNFSF ligand expression, we confirmed previous reports on IAV-induced upregulation of *Tnfsf10* in lung macrophages (25, 26), whereas *Tnfsf15* and *Tnfsf14* were moderately increased (Figure 3F and Supplemental Data 1, TNF signaling arrays WT data). Overall, severe IAV infection was linked to a total TNFSF14 increase in the mouse lung, as shown by IHC (Figure 3G), quantitative PCR (qPCR) (Figure 3H), and ELISA (Figure 3I). In accordance with this finding, we observed a significant increase in soluble TNFSF14 in the BALF of patients with influenza or COVID-19 acute respiratory distress syndrome (ARDS) compared with control patients who underwent routine bronchoscopy for diagnostic purposes and had normal BALF cellularity (Figure 3J). On the basis of the distinct kinetics of the two TNFSF14 receptors, we aimed to dissect any differential roles in post-influenza TR-AM fate. Anti-LTβR TR-AM pretreatment significantly attenuated the increase in caspase 3/-7 activity after iBALF treatment, as opposed to that observed in the anti-TNFRSF14 and isotype control groups (Figure 3K). Similar results were observed when we treated naive WT, *Tnfrsf14^–/–^*, and *Ltbr^–/–^* TR-AMs with iBALF (Figure 3L), indicating a potential protective effect against soluble death-inducing ligands in the absence of LTβR. In accordance with this finding, IAV-infected *Ltbr^–/–^* mice showed a less dramatic drop in TR-AM numbers compared with WT and *Tnfrsf14^–/–^* mice (Figure 3M), as well as an overall attenuated weight loss on days 7 and 8 p.i. (Figure 3N). The distinct effects of the 2 receptors on TR-AM survival and the upregulation of the common TNFSF14 ligand in IAV-infected lungs led us to more closely examine TNFSF14.

### TNFSF14 drives the depletion of the TR-AM pool in IAV-induced pneumonia.

**Figure 4:**
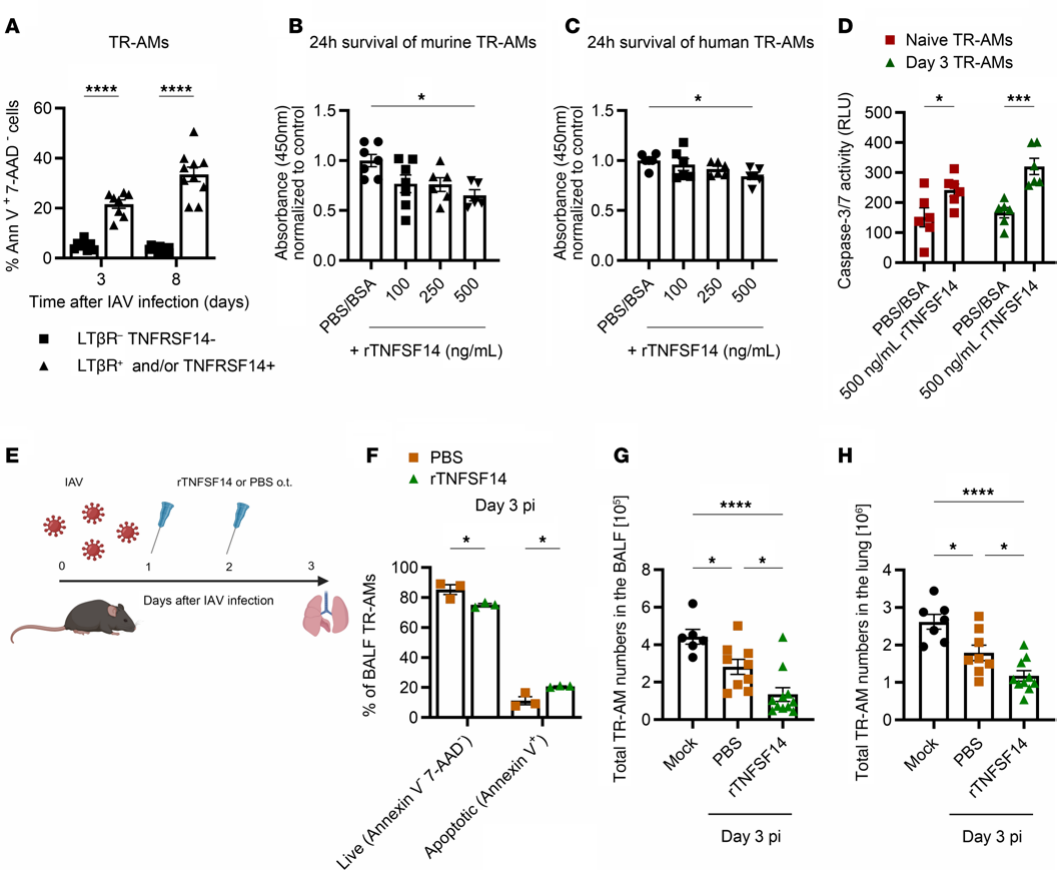
TNFSF14 treatment aggravates post-influenza TR-AM loss. (**A**) Percentage of apoptotic cells within LTβR^–^TNFRSF14^–^ and LTβR^+^ and/or TNFRSF14^+^ TR-AM subgroups on days 3 and 8 p.i. (*n* = 8–10 per group; data represent the mean ± SEM and were pooled from 2 independent experiments). (**B** and **C**) Cell survival proportional to light absorbance after a 24-hour treatment of murine (**B**) and human (**C**) TR-AMs with different doses of rTNFSF14 and normalized to control samples (*n* = 5–7; data represent the mean ± SEM and were pooled from 7 and 4 independent experiments in **B** and **C**, respectively). (**D**) Caspase 3/-7 activity after 24 hours of TR-AM treatment with 500 ng/mL rTNFSF14 (*n* = 6–7; data represent the mean ± SEM and were pooled from 6 independent experiments). (**E**) Schematics of rTNFSF14 application to IAV-infected mice on days 1 and 2 p.i., with analysis performed on day 3 p.i. Figure created with BioRender.com. (**F**) Percentage of live (annexin V^–^ 7-AAD^–^) and apoptotic (annexin V^+^7-AAD^–^) TR-AMs (*n* = 3) on day 3 after rTNFSF14 treatment, data are representative of 3 different experiments. (**G** and **H**) Total BALF (*n* = 6–11, **G**) and lung-tissue TR-AMs (*n* = 7–10, **H**) on day 3 following rTNFSF14 treatment compared with mock infection. Data represent the mean ± SEM and were pooled from 7 independent experiments. *P < 0.05, ***P < 0.001, and ****P < 0.0001, by 1-way ANOVA with Tukey’s post hoc test (**B**, **C**, **G**, and **H**) and 2-way ANOVA with Tukey’s post hoc test (**A**, **D**, and **F**).

To test whether TNFSF14 only induced apoptosis in a subpopulation of TR-AMs, TNFSF14 receptor expression on the surface of TR-AMs was combined with apoptosis staining (annexin V/7-aminoactinomycin [7-AAD]) at different time points after infection. TNFSF14 receptor–expressing cells comprised approximately 80% of apoptotic TR-AMs on day 3 p.i. (Supplemental Figure 4B). When we compared TR-AM apoptosis induction based on TNFSF14 receptor expression, we found that TNFSF14 receptor–positive TR-AMs had significantly higher apoptosis rates on days 3 and 8 p.i. compared with LTβR^–^TNFRSF14^–^ cells (Figure 4A). To test whether TNFSF14 could directly induce TR-AM death, we treated naive murine (Figure 4B) and human BALF TR-AMs (Figure 4C) with different concentrations of recombinant TNFSF14 (rTNFSF14) for 24 hours. Treatment with 500 ng/mL rTNFSF14 led to an average of 25%–35% decrease in TR-AM survival compared with the control PBS/BSA-treated group (Figure 4, B and C) and an increase in caspase 3/-7 activity, which was even more pronounced when the same experiment was performed with day 3 TR-AMs (Figure 4D). Unlike TR-AMs, epithelial cells, which presented a basolateral LTβR and a nonpreferential, cytoplasmic TNFRSF14 expression pattern (Supplemental Figure 4, D–G) and were also exposed to homotrimeric, active (27) TNFSF14 in the BALF (Supplemental Figure 4, H and I), did not succumb to TNFSF14-induced apoptosis (Supplemental Figure 4C), mirroring the diverse roles of TNFSF14 signaling, based on cell type and receptor availability.

**Figure 5:**
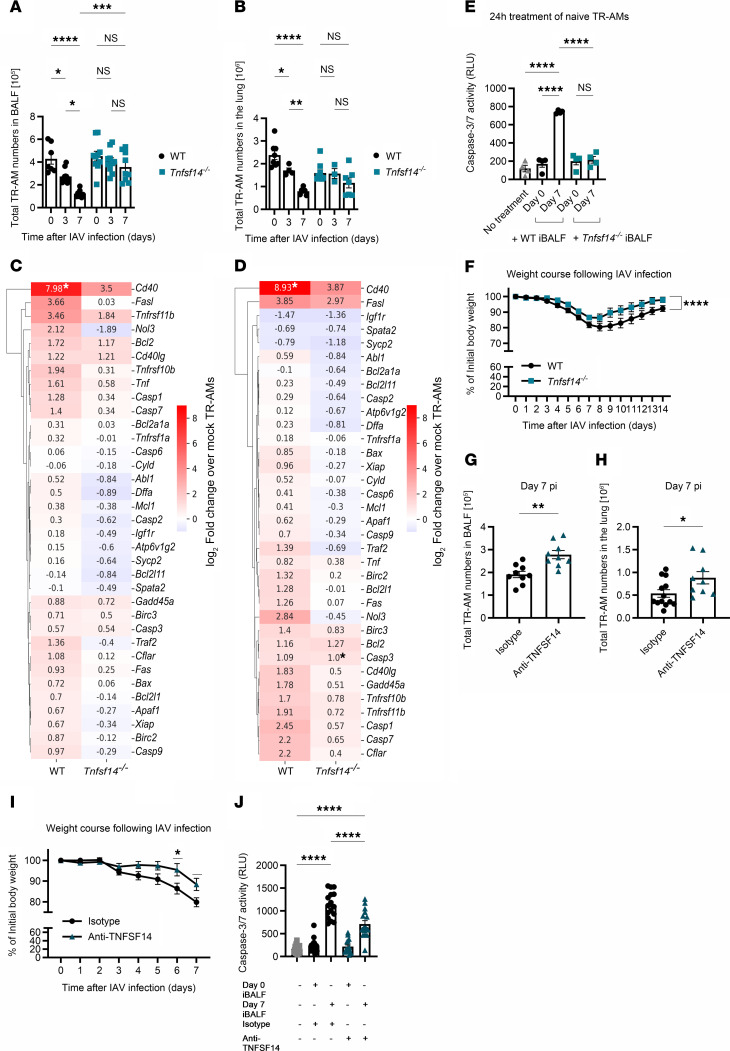
Post-influenza TR-AM loss can be prevented through directed targeting of the TNFSF14 ligand. (**A** and **B**) BALF TR-AMs (**A**) and sessile lung TR-AMs (**B**) from WT and *Tnfsf14^–/–^* mice after IAV infection (*n* = 8–12; data represent the mean ± SEM and were pooled from 17 different experiments; WT controls in **B**, including values, are depicted in Supplemental Figure 2A). (**C** and **D**) Heatmaps depicting the fold change of apoptosis-related genes on day 3 p.i. (**C**) and day 7 p.i. (**D**) over mock-infected WT and *Tnfsf14^–/–^* TR-AMs (*n* = 3–5; data were pooled from 5 different experiments; WT were data extracted from the dataset presented in Figure 2E). (**E**) Caspase 3/-7 activity after treatment of TR-AMs with WT or *Tnfsf14^–/–^* iBALF (*n* = 4 per condition; data represent the mean ± SEM and are representative of 3 independent experiments). (**F**) Body weight of WT and *Tnfsf14^–/–^* mice over the IAV infection course (*n* = 12–13; data represent the mean ± SEM of weight at each time point and were pooled from 6 independent experiments; WT controls including data are presented in Figure 3M). (**G** and **H**) BALF (**G**) and lung tissue (**H**) TR-AMs on day 7 after anti-TNFSF14 treatment on day 2 p.i. (*n* = 9–13; data represent the mean ± SEM and were pooled from 4 independent experiments). (**I**) Body weight of anti-TNFSF14 and isotype-treated WT mice after IAV infection ( *n* = 10–11; data represent the mean ± SEM of weight at each time point and were pooled from 4 independent experiments). (**J**) Caspase 3/-7 activity after 24 hours of iBALF treatment of anti-TNFSF14–pretreated naive TR-AMs (*n* = 16; data represent the mean ± SEM and were pooled from 6 independent experiments). *P < 0.05, **P < 0.01, ***P < 0.001, and ****P < 0.0001. Multiple unpaired, 2-tailed Student’s *t* tests for weight-loss comparisons at each time point after infection (**G**–**I**); 2-tailed Student’s *t* test for the log_2_ fold-change values of each gene in the compared groups (**C** and **D**); 1-way ANOVA with Tukey’s post hoc test (**E** and **J**), and 2-way ANOVA with Tukey’s post hoc test (**A** and **B**).

Application of rTNFSF14 (o.t.) in IAV-infected mice (Figure 4E) led to a significant increase in the number of annexin V^+^ TR-AMs (Figure 4F) and further reduced the already diminished BALF and lavaged lung tissue TR-AM numbers on day 3 p.i. (Figure 4, G and H) compared with PBS-treated, IAV-infected controls. No differences could be detected in the numbers of other leukocyte populations, based on rTNFSF14 treatment (Supplemental Figure 5, A–E). Concomitantly, TR-AM loss was completely abrogated in the BALF and lavaged lung tissue of *Tnfsf14^–/–^* mice (Figure 5, A and B). This result could not be reproduced in *Tnfsf10^–/–^* mice (Supplemental Figure 5F), despite TNFSF10 being one of the proapoptotic ligands highly expressed in BALF after IAV infection (25, 26), suggesting a ligand-specific induction of TR-AM apoptosis. Flow-sorted *Tnfsf14^–/–^* TR-AMs showed lower induction of apoptosis, necrosis, and autophagy-related gene expression (Figure 5, C and D, and Supplemental Figure 5, G–J, Supplemental Data 3, cell death arrays *Tnfsf14*-KO data) on days 3 and 7 p.i. compared with WT mice. Unlike WT iBALF, caspase 3/-7 activity was not increased upon TR-AM treatment with *Tnfsf14^–/–^* iBALF (Figure 5E). No differences could be detected in viral titers on day 3 p.i. (peak of viral replication in this model [ref. 28], Supplemental Figure 5K) or in the amount of epithelial (EpcAM^+^), endothelial (CD31^+^), and mesenchymal cells (MCs) (Supplemental Figure 5, L–N). With the exception of neutrophils, which were found in higher numbers in *Tnfsf14^–/–^* mice on day 3 p.i. but not at later time points (Supplemental Figure 5O), we observed no differences for other BALF leukocyte populations (Supplemental Figure 5, P–S). These data suggested that TNFSF14-associated cell death was confined to the TR-AM compartment. Alongside this finding, absence of the ligand resulted in decreased weight loss, hinting at a beneficial effect of TNFSF14 blockade (Figure 5F). With the aim of applying a therapeutic approach, we used a neutralizing anti-TNFSF14 antibody (11) and observed higher BALF and lung tissue TR-AM numbers on day 7 p.i. (Figure 5, G and H), an attenuated weight loss in the anti-TNFSF14 group (Figure 5I), and confirmed lower caspase 3 activity in TR-AMs after ex vivo iBALF treatment (Figure 5J), highlighting TNFSF14 as the driver of post-influenza TR-AM death.

### TNFSF14 is released by neutrophils during the acute IAV infection phase.

**Figure 6:**
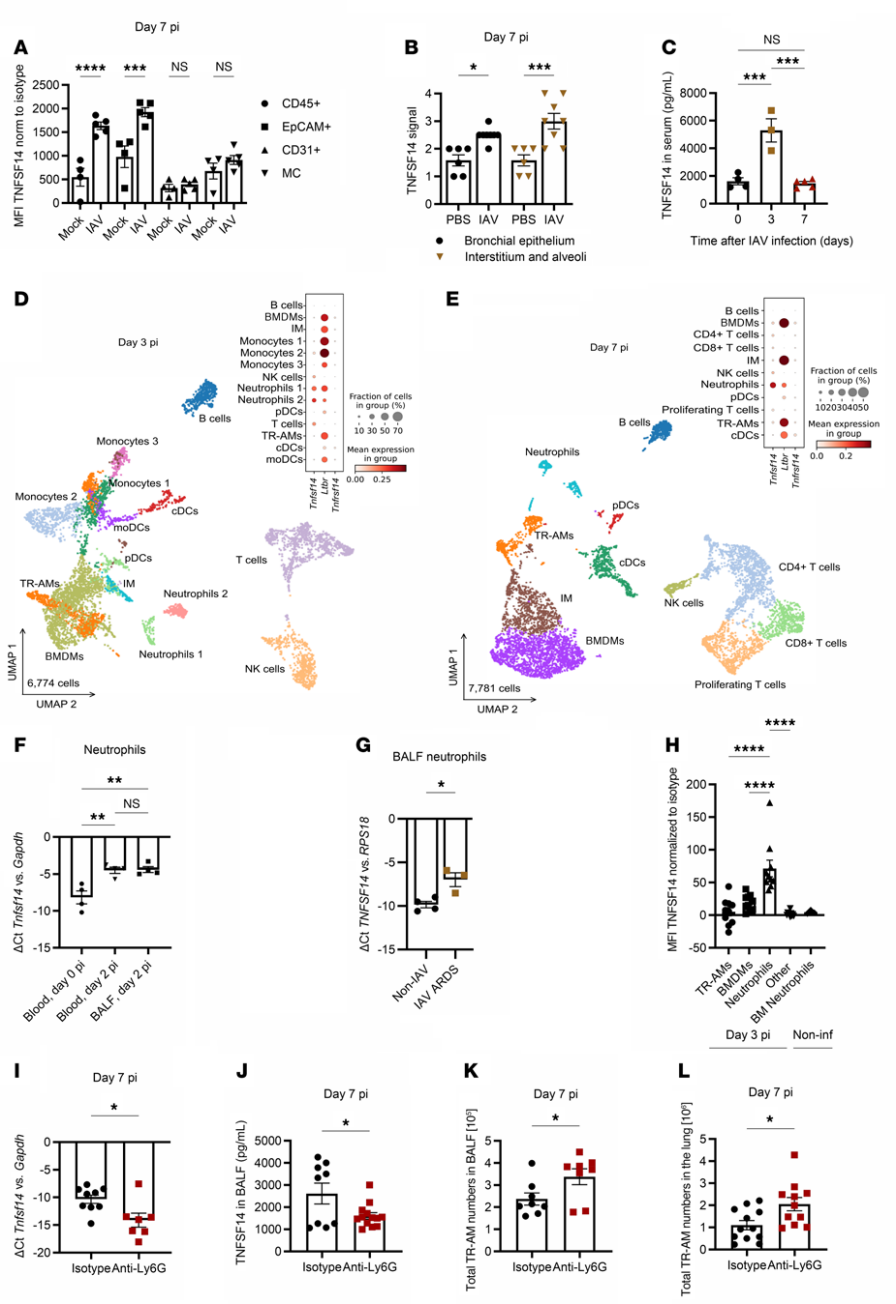
Neutrophils are the main cellular source of TNFSF14 during IAV infection. (**A**) TNFSF14 expression on leukocytes, epithelial cells, endothelial cells, and MCs in the lungs of noninfected and day 7–infected mice (*n* = 4–5, were data pooled from 2 independent experiments). (**B**) TNFSF14 expression in different lung regions of mock- and IAV-infected WT mice on day 7 p.i. (*n* = 3–4; data are representative of 2 independent experiments). (**C**) Serum TNFSF14 levels on days 0, 3, and 7 p.i. (*n* = 3–5; data were pooled from 3 independent experiments). (**D** and **E**) Leukocyte scRNA-Seq analysis on day 3 p.i. (**D**) and day 7 p.i. (**E**) (*n* = 4; data are from 2 independent experiments). Monocyte and neutrophil clusters on day 3 p.i. were characterized according to the gene signature of the top 5 uniquely expressed genes per cluster. Dot plots depict *Tnfsf14*, *Ltbr*, and *Tnfrsf14* expression. (**F**) qPCR analysis of *Tnfsf14* expression in blood and BALF neutrophils (*n* = 4; data were pooled from 2 independent experiments). (**G**) qPCR analysisof *TNFSF14* expression in BALF neutrophils from patients with severe IAV-induced ARDS compared with non-IAV controls (*n* = 3–4). (**H**) TNFSF14 expression on BALF leukocytes on day 3 p.i. and bone marrow–derived neubrophils (BM Neutrophils) from noninfected mice (*n* = 3–10; data were pooled from 2 independent experiments). (**I** and **J**) qPCR analysis in lung tissue (*n* = 7–9, **I**) and BALF ELISA (*n* = 9–13, **J**) for TNFSF14 expression on day 7 after neutrophil depletion. Data were pooled from 4 and 5 independent experiments, respectively. (**K** and **L**) BALF (**K**) and lung (**L**) TR-AMs on day 7 after neutrophil depletion (*n* = 8; data represent the mean ± SEM and were pooled from 4 and 5 independent experiments, respectively). *P < 0.05, **P < 0.01, ***P < 0.001, and ****P < 0.0001, by unpaired, 2-tailed Student’s *t* test (**G** and **L**), 1-way ANOVA with Tukey’s post hoc test (**C**, **F**, and **H**), and 2-way ANOVA with Tukey’s post hoc test (**A** and **B**).

To identify the cellular source of TNFSF14 during IAV pneumonia, we analyzed expression levels of the transmembrane form of TNFSF14 on leukocytes (CD45^+^ cells), epithelial cells, MCs, and endothelial cells in the lungs of mock- and IAV-infected mice on day 7 p.i. We found that TNFSF14 expression was significantly increased in epithelial cells and leukocytes (Figure 6A). This was in accordance with our IHC data, which revealed a prominent signal increase within the leukocyte-infiltrated interstitium and alveolar space on day 7 p.i. (Figure 6B). Soluble TNFSF14 was detected in the serum of infected mice as early as day 3 p.i., further suggesting that blood-derived immune cells contributed to the increase in TNFSF14 levels in the lungs (Figure 6C). Single-cell RNA-Seq (scRNA-Seq) analysis of pregated CD45^+^ cells from whole lung digests on days 3 and 7 p.i. revealed a total of 14 immune cell clusters on day 3 p.i., including TR-AMs, BMDMs, interstitial macrophages (IMs), B and T cells, conventional DCs (cDCs), plasmacytoid DCs (pDCs), monocyte-derived DCs (moDCs), NK cells, 3 monocyte clusters with different gene signatures (monocytes 1: *Ccr2*, *Ly6a2*, *F13a1*, *Mgst1*, *Aldh2*; monocytes 2: *Tgfb1*, *Sirpb1c*, *Otulin1*, *Plcg2*, *Zfp710*; and monocytes 3: *Eno3*, *Cd300e*, *Agpat4*, *Rbpms*, *Slc12a2*), and 2 neutrophil clusters (neutrophils 1: *Ier5*, *Ier3*, *Smox*, *Gm8995*, *Ccrl2*; and neutrophils 2: *Picalm*, *Jund*, *Hmgb2*, *Map1lc3b*, *Slc2a3*). Eleven clusters were identified on day 7 p.i., including TR-AMs, BMDMs, IMs, B cells, CD4^+^ and CD8^+^ T cells, proliferating T cells, pDCs, cDCs, NK cells, and neutrophils (Figure 6, D and E). At both time points, neutrophils were revealed to be the main leukocyte population expressing *Tnfsf14*, with a minor contribution from NK and T cells. *Ltbr* gene expression was higher than that of *Tnfrsf14* in all monocyte/macrophage populations, including TR-AMs (Figure 6, D and E). qPCR analysis revealed upregulation of *Tnfsf14* in neutrophils isolated from peripheral blood on day 2 p.i. (Figure 6F), which was recapitulated in BALF neutrophils of patients with IAV ARDS (Figure 6G). Flow cytometric analysis confirmed neutrophils as the main TNFSF14-expressing leukocyte population on day 3 p.i., with no ligand expression on bone marrow–derived neutrophils from noninfected mice (Figure 6H). Both soluble and transmembrane TNFSF14 were revealed to contribute to TR-AM apoptosis, as iBALF treatment of day 3 TR-AMs led to a significant increase in caspase 3/-7 activity, comparable to the increase induced by TR-AM coculture with day 3 neutrophils, even in the presence of a metalloproteinase inhibitor preventing TNFSF14 shedding (Supplemental Figure 6A) (29). We then tested whether neutrophil depletion via i.p. administration of an anti-Ly6G antibody (30) would improve post-influenza TR-AM survival. Following successful neutrophil depletion (Supplemental Figure 6, B–E), the mice had attenuated weight loss up to day 7 p.i. (Supplemental Figure 6F), which was in accordance with previously published data (31, 32). Neutrophil-depleted mice had lower TNFSF14 levels (Figure 6, I and J) compared with levels in the isotype-treated group, with higher BALF and lung-tissue TR-AM numbers on day 7 p.i. (Figure 6, K and L). We observed no significant differences in other BALF immune cells (Supplemental Figure 6, G–K), indicating the depletion specificity of the anti-Ly6G antibody.

### Outcome of post-influenza pneumococcal pneumonia is improved by targeting the TNFSF14 ligand/receptor axis.

**Figure 7:**
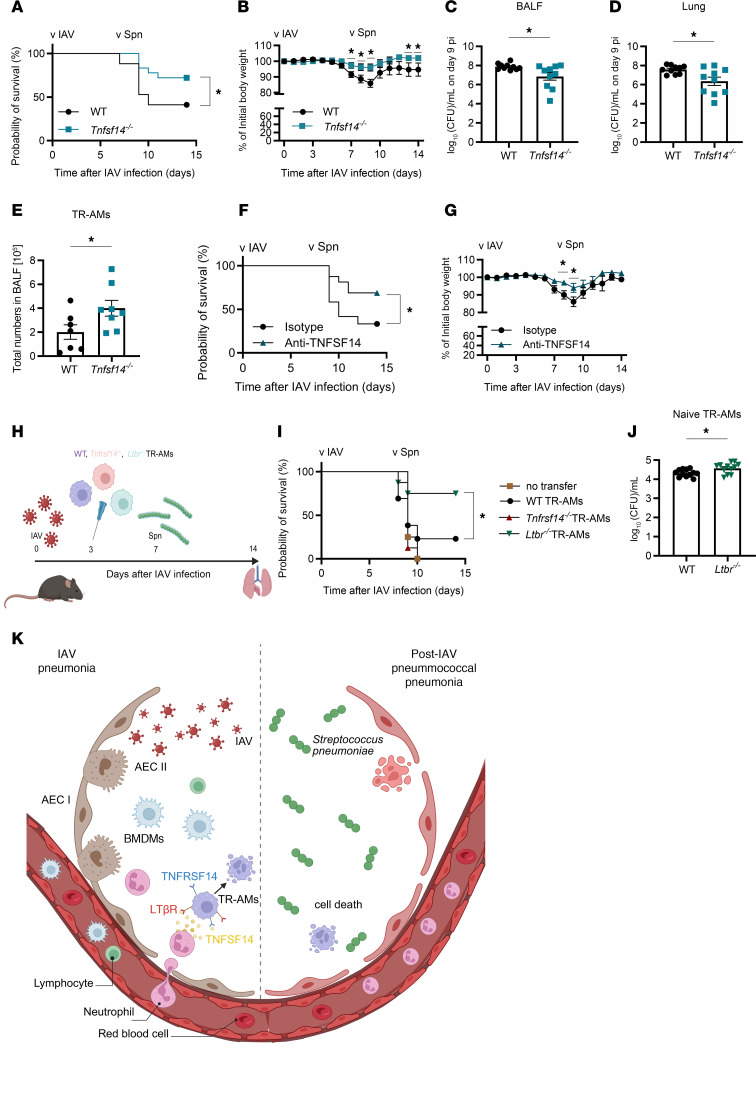
Severity of post-influenza pneumococcal pneumonia is attenuated in the absence of TNFSF14. (**A** and **B**) Survival (**A**) and weight loss (**B**) after IAV and *S. pneumoniae* coinfection of WT and *Tnfsf14^–/–^* mice (*n* = 17–18; data represent the mean ± SEM and were pooled from 5 different experiments). (**C** and **D**) Bacterial burden in the BALF (**C**) and lungs (**D**) of WT and *Tnfsf14^–/–^* mice 9 days after IAV infection and 48 hours after *S. pneumoniae* infection (*n* = 10; data represent the mean ± SEM and were pooled from 8 independent experiments). (**E**) Total BALF TR-AM numbers for WT and *Tnfsf14^–/–^* mice 9 days after IAV and 48 hours after *S. pneumoniae* infection (*n* = 7–8; data represent the mean ± SEM and were pooled from 5 different experiments). (**F** and **G**) Survival (**F**) and weight loss (**G**) following IAV and *S. pneumoniae* coinfection and TNFSF14 blocking on day 2 p.i. (*n* = 12–16; data represent the mean ± SEM and were pooled from 5 independent experiments). (**H**) Schematics of the experimental layout for IAV and *S. pneumoniae* coinfection with adoptive transfer of naive WT, *Tnfrsf14^–/–^*, or *Ltbr^–/–^* TR-AMs on day 3 p.i. (**I**) Survival of WT mice upon IAV and *S. pneumoniae* coinfection and TR-AM adoptive transfer (*n* = 4–12; data represent the mean ± SEM and were pooled from 5 independent experiments). **(J)** Bacterial load in lysed WT and *Ltbr^–/–^* naive TR-AMs after ex vivo *S. pneumoniae* infection (*n* = 12; data represent the mean ± SEM and were pooled from 4 independent experiments). *P < 0.05 and **P < 0.01, by log-rank (Mantel-Cox) test (**A**, **F**, and **I**); unpaired, 2-tailed Student’s *t* test (**B** and **G**). Multiple unpaired Student’s *t* tests were performed for weight-loss comparisons at each time point after infection (**C**, **D**, **E**, and **J**). (**K**) Proposed hypothesis: Severe IAV–induced pneumonia is characterized by massive leukocyte recruitment, including neutrophils. Once in the alveoli, neutrophils start releasing TNFSF14, which is sensed by TR-AMs through ligation to the surface-expressed receptors TNFRSF14 and LTβR, culminating in TR-AM death, which increases host susceptibility to post-influenza pneumococcal pneumonia. Panels **H** and **K** were created with BioRender.com.

Having established that TR-AM loss was driven by the TNFSF14/LTβR axis in severe IAV infection, we tested whether TR-AM preservation would improve coinfection outcomes (Figure 1, A–C). Indeed, *Tnfsf14^–/–^* mice showed significantly improved survival and reduced weight loss (Figure 7, A and B), and reduced BALF pneumococcal burden on day 9 p.i. and 48 hours after *S. pneumoniae* infection (Figure 7, C and D), whereas no difference was seen for bacterial burden in the spleen (Supplemental Figure 7A). Despite a massive neutrophil influx in both groups (Supplemental Figure 7B), *Tnfsf14^–/–^* mice maintained TR-AM numbers (Figure 7E). No significant differences were observed in the phagocytic capacity or the percentage of phagocytic TR-AMs on day 7 p.i., the time point of pneumococcal infection (Supplemental Figure 7, C and D). Given the lack of difference in overall phagocytic capacity, we concluded that the improved bacterial clearance in *Tnfsf14^–/–^* mice was mainly attributed to the higher number of surviving TR-AMs. Treatment with the neutralizing anti-TNFSF14 antibody led to a similar improvement in survival and weight loss (Figure 7, F and G), supporting the hypothesis that post-influenza TR-AM maintenance is key to survive secondary pneumococcal infection. Finally, we performed orthotopic transfer of WT, *Tnfrsf14^–/–^*, or *Ltbr^–/–^* TR-AMs in coinfected mice (schematic in Figure 7H). Pneumococcal superinfection proved lethal in mice that received no cells or *Tnfrsf14^–/–^* TR-AMs, whereas transfer of *Ltbr^–/–^* TR-AMs rescued 75% of the mice (Figure 7I). Transfer of WT TR-AMs only slightly increased survival, indicating that transferred WT TR-AMs underwent TNFSF14-dependent apoptosis after transfer on day 3 p.i. *Ltbr^–/–^* TR-AMs additionally showed a superior phagocytic capacity for *S. pneumoniae* compared with WT TR-AMs when infected ex vivo (Figure 7J), which may have additionally contributed to the improved survival observed in the *Ltbr^–/–^* TR-AM recipient group. On the basis of these results, we conclude that targeting the TNFSF14 signaling axis could revert TR-AM death in the aftermath of severe IAV-induced lung injury and thus prevent the transition to secondary bacterial pneumonia (Figure 7K).

## Discussion

Lower respiratory tract infections (LRTIs) are a leading cause of death globally, with IAV infection playing a major role due to a variety of potential complications, most notably secondary bacterial infections, which greatly increase the risk of respiratory failure and ICU admission as well as overall mortality (21, 33, 34). With no causative pharmacological treatment for pneumonia-related lung injury, research has focused on understanding the mechanisms behind severe IAV pneumonia and the transition to post-influenza secondary infection. Proposed mechanisms include bacterial dissemination due to IAV-associated epithelial cell death, fibrin deposition, impaired mechanical clearance, microbial dysbiosis, and IFN-driven suppression of phagocyte function (2, 35, 36). As the lungs’ first line of defense, IAV-induced TR-AM depletion is a critical step in compromising host immunity. Patient and animal studies have demonstrated that severe viral infections drive TR-AM depletion and niche replenishment by BMDMs, with the depletion phase aligning with peak susceptibility to bacterial infection (3, 37–39), yet the involved pathways remain poorly understood. Here, we identified TNFSF14 as a driver of TR-AM loss during IAV pneumonia.

In the first week after infection, TR-AM numbers progressively declined, while other leukocytes gradually entered the alveoli in response to viral infection. TR-AM fate after acute infection is dictated by cell death–inducing mechanisms, impaired self-renewal capacity, and loss of prosurvival signals from the injured neighboring epithelium (6, 7). The extent of TR-AM depletion and the intensity of the inflammatory response shape the composition and (re)programming of lung-resident cells after infection (40). Our own previous data indicate that partial TR-AM loss enables the recruitment of circulating BMDMs, which are essential for post-viral repair through their transitioning into prohomeostatic phenotypes (41). Coexistence of newly recruited BMDMs and surviving original TR-AMs is the outcome of a balanced immune response, which culminates in BMDM-orchestrated tissue repair and return to homeostasis, assisted by the tolerogenic functions of TR-AMs, aimed at restricting epithelial damage (42, 43). Infection severity determines the extent of TR-AM depletion, with a dramatic loss upon severe IAV pneumonia, as demonstrated in our model. Recruitment of proinflammatory immune cells and chemokine abundance contribute to viral clearance but can also escalate to an unbalanced immune response (44). The highly proinflammatory programming of BMDMs can aggravate local injury and promote aberrant lung remodeling (18, 28), while dysregulated neutrophil migration and activation positively correlate with disease severity and poor patient outcomes (45, 46). Following TR-AM depletion, early recruitment of professional phagocytes such as BMDMs and neutrophils failed to control bacterial spread, leading to dramatic bacterial outgrowth within 24 hours of *S. pneumoniae* infection. This aligns with prior studies demonstrating high susceptibility to secondary pneumococcal infection 5–7 days after IAV infection, coinciding with the TR-AM depletion phase (3). IFN-γ, which is profusely released in the alveoli as part of the antiviral response, heavily impairs TR-AM antibacterial properties, as it downregulates the macrophage receptor with collagenous structure scavenger receptor (MARCO) on the surface of TR-AMs, one of the key elements in the TR-AM antibacterial response (47, 48). Defective chemokine production by macrophages, as observed in severe IAV infection and sepsis models, further aggravates the disease outcome (49, 50). The near-complete TR-AM loss within a microenvironment of abundant death-inducing signals in our model highlights an additional important step toward the establishment of lethal post-viral pneumococcal pneumonia. Although a considerable advantage in terms of antibacterial properties has been described for infection-experienced BMDMs and newly originating, BMDM-derived TR-AMs after IAV infection (19), pneumococcal infection in that model was performed weeks after the initial viral hit, as opposed to the window of increased host vulnerability during the acute infection phase described in our study. At this point, TR-AMs showed superior phagocytic capacity and a killing capacity similar to that of BMDMs. Thus, preserving TR-AMs early on may provide critical protection until reestablishment of a fully functional resident macrophage niche, including infection-trained BMDMs, has been completed.

The remarkable TR-AM loss in WT C57BL/6 mice in our study differs from previously published data, in which the mouse’s genetic strain determined TR-AM survival, with BALB/c mice exhibiting a drastic TR-AM reduction, unlike C57BL/6 mice, which maintained TR-AMs of an altered phenotype (51). In this study by Califano et al., a relatively low dose of IAV PR8 was administered i.n., whereas in our study, we administered a high viral dose via the o.t. route, with the aim of inducing severe pneumonia. The animal strain and administration route for the infection may, therefore, depict a limitation of our study, as results may differ for different in vivo models.

Transcriptomics and flow cytometric analyses revealed apoptosis as the primary cause of post-influenza TR-AM death, largely independent of direct viral infection, suggesting the involvement of a death-inducing ligand. While apoptosis promotes early viral spread (22, 52), it also limits infection through elimination of infected cells (53, 54). Leukocyte- and virus-driven AEC apoptosis, however, compromises the gas-blood barrier and impairs gas exchange (25, 28). Apoptosis inhibition can, therefore, influence the infection outcome. To compensate for any effect of caspase inhibition on early virus propagation, we began our treatment on day 2 p.i. and observed no significant difference in viral titers on day 3 p.i., the peak of viral replication in this model (28). Caspase 8 inhibition was chosen on the basis of our in vitro data, which showed a clear advantage over caspase 3 inhibition after TR-AM iBALF treatment. This initially surprising result could be potentially explained through PANoptosis as the joint result of pyroptosis, apoptosis, and necroptosis. This would permit cell death via caspase 3–independent apoptosis or pyroptosis. Caspase 8 is involved in both of these pathways (55–58) and is currently the only known programmed cell death (PCD) member that connects all PCD pathways. This can explain the complete abrogation of TR-AM death on day 3 p.i., the reduced weight loss, and the improved TR-AM survival upon caspase 8 inhibition. Nevertheless, the PANoptosis concept suggests that a single PCD component cannot individually rescue cells once PANoptosis has been initiated, which might explain why TR-AM loss was not completely prevented on day 7 p.i., when death-inducing signals are highly abundant (59–61).

Stochastic interrogation of TNFSF members revealed substantial upregulation of TNFSF14 in infected mouse lungs, with high soluble TNFSF14 levels also found in BALF from patients with severe virus-induced ARDS. Previous studies on severe viral pneumonia and sepsis positively linked elevated BALF/serum TNFSF14 levels to disease severity (15, 16, 62, 63). Depending on the cell type, pathogen interaction, and receptor availability, TNFSF14 can influence cell survival, (re)programming of the cell profile, immune response establishment, and infection memory (10, 64). TNFSF14 has been previously described as a determinant of macrophage survival, phenotype, and antibacterial properties (65–67), however, extensive studies regarding post-influenza TR-AM death are lacking. In our study, TNFSF14 deletion or blockade preserved TR-AMs and improved survival and weight loss during coinfection. These benefits were not solely due to the reduced bacterial burden but likely stemmed from enhanced tissue repair and an accelerated return to homeostasis due to improved TR-AM survival. Previous work from our laboratory highlighted TR-AMs as drivers of epithelial repair (41) and mitigators of lung inflammation, even at the cost of bacterial clearance (43). Further experiments would be required to fully address the role of TNFSF14 in pathogen resistance and tolerance in the context of coinfection beyond the lung-confined effect on TR-AM survival.

We found that post-influenza TNFSF14-induced TR-AM death was cell specific, with no significant differences in other leukocytes (except neutrophils on day 3 p.i.) or in endothelial, mesenchymal, or epithelial cells between WT and *Tnfsf14^–/–^* mice. TNFSF14 treatment did not worsen virus-induced death in AECs, suggesting that TNFSF14 was not a strong driver of post-influenza distal epithelial cell apoptosis. We identified neutrophils as the main leukocyte source of TNFSF14, which is in accordance with previously published data (68, 69). TNFSF14 has been shown to play an instrumental role in NK and T cell activation and expansion (11, 17, 70) and DC maturation (71) and may thus serve as an intermediate between the acute and adaptive immune response. Aberrant release due to dysregulated neutrophil activation could offer an alternative explanation for the abundance of TNFSF14 upon severe infection. This is in accordance with literature, as high circulating or organ-specific TNFSF14 levels have been positively correlated with highly inflammatory states (13, 15, 72–74), and blocking of the ligand was shown to limit inflammation and attenuate organ injury (75).

TNFRSF14 and LTβR, the 2 competitive TNFSF14 receptors, followed distinct kinetics in terms of transcriptional regulation and protein expression in TR-AMs during the infection course. Attenuated TR-AM loss after IAV infection of *Ltbr^–/–^* mice, compared with *Tnfrsf14^–/–^* mice, and improved survival after intrapulmonary transfer of *Ltbr^–/–^* TR-AMs to coinfected WT mice demonstrated a stronger effect on TR-AM death for LTβR. Given the more prominent TR-AM preservation in *Tnfsf14^–/–^* mice compared with *Ltbr^–/–^* mice, we hypothesize that cell death could also be initiated through ligation of TNFSF14 to TNFRSF14, potentially through activation of a coreceptor, as TNFRSF14 lacks a pro-death domain (10, 76). It should be noted, however, that engagement of LTβR by TNFSF14 on macrophages is not merely confined to apoptosis induction. TGF-β can be secreted upon crosslinking (77), which has been shown to drive an immunoparalysis state in the aftermath of infection, further enhancing secondary infection susceptibility (78). LTβR-TNFSF14 interaction on the endothelium alters microvasculature structure, which can in turn favor the recruitment of immune cells (79). The intricate nature of TNFSF14-TNFRSF14-LTβR interactions thus points to a multitude of potential roles for the signaling axis in the context of influenza, besides TR-AM depletion. Nevertheless, with clinical trials in the context of virus-induced pneumonia and systemic inflammation already revealing beneficial safety profiles (63, 80, 81), therapeutic interventions disrupting TNFSF14-initiated intercellular pathways to preserve TR-AM function appear to be promising approaches for improving host defense in the context of IAV pneumonia.

## Methods

### Sex as a biological variable.

Sex was not considered as a biological variable for patient samples. Both male and female mice were used for all studies.

### Mice.

Wt C57BL/6 mice were purchased from Charles River Laboratories. *Tnfsf14^–/–^* (82), *Tnfrsf14^–/–^* (83), and *Ltbr^–/–^* (84) mice were a gift from Klaus Pfeffer (Heinrich Heine University Düsseldorf, Düsseldorf, Germany). *Tnfsf10^–/–^* (85) mice were obtained from AMGen. All mice were bred under specific pathogen–free (SPF) conditions and infected at 10–12 weeks of age.

### In vivo infection.

For in vivo IAV infection experiments, mice were o.t. inoculated with 250–1,000 FFU A/Puerto Rico/8/1934 (PR8, H1N1) influenza virus. Control groups were inoculated with sterile PBS^–/–^. For coinfection experiments, mice were i.n. infected with 20 CFU *S. pneumoniae* (serotype 3, strain PN36 [NCTC 7978]), provided by M. Witzenrath and co-workers (Department of Infectious Diseases and Pulmonary Medicine, Charité, University Medicine Berlin, Berlin, Germany) 7 days after IAV infection.

### In vivo treatment.

For apoptosis inhibition, WT mice were infected with 500 FFU (day 7 experiments) or 1,000 FFU IAV (day 3 experiments) and treated with s.c. injections of 10 mg/kg Z-IETD-FMK (R&D Systems), a specific caspase 8 inhibitor, or a DMSO control. For day 3 experiments, treatment involved a single injection on day 2 p.i., whereas daily injections were applied on days 2–6 p.i. for analysis on day 7 p.i. Neutrophil depletion was performed through i.p. application of 200 μg anti-1A8 antibody (InVivoPlus rat anti–mouse Ly6G, catalog BP0075-1, BioXCell) or anti-2A3 isotype control (InVivoPlus rat IgG2a isotype control, anti-trinitrophenol, catalog BP0089, BioXCell) diluted in sterile PBS in mice infected with 500 FFU IAV on days –1, 1, 3, and 5 p.i. TNFSF14 neutralization was achieved with a mouse anti–mouse TNFSF14 blocking antibody (clone 3D11, IgG2b, k, isotype control mouse IgG2b, clone 27–35, BioLegend), provided by José Ignacio Rodríguez Barbosa and Maria-Luisa del Rio (INBIOMIC, University of León, León, Spain). A single i.p. injection of 500 μg antibody or isotype control was performed 2 days after IAV infection with 500 FFU. For in vivo treatment with rTNFSF14, 10 μg carrier-free mouse rTNFSF14 (cataglo 1794-LT, R&D Systems) diluted in 0.03 mL PBS^–/–^ was o.t. administered to IAV-infected mice on days 1 and 2 p.i. for analysis on day 3 p.i.

### Adoptive TR-AM transfer.

Murine TR-AMs were obtained from the BALF of naive WT, *Tnfrsf14^–/–^,* and *Ltbr^–/–^* mice, as previously described (25). Adoptive transfer of 400,000 TR-AMs per mouse was performed on day 3 p.i. of WT mice with 250 FFU IAV, with an engraftment efficiency of 14%–20% calculated 24 hours later, at a time point where over 70% of the original TR-AM pool was still detectable in the BALF of infected mice (data not shown). Secondary pneumococcal infection was performed 4 days later.

### TR-AM isolation and cell culture for ex vivo treatment.

Following BALF extraction from naive mice, cells were resuspended in full TR-AM medium (RPMI-1640/2% FBS/2.5% HEPES/1% l-glutamine/1% penicillin-streptomycin). TR-AMs were seeded at a density of 10,000–50,000 cells/well on a 96-well plate.

### Colorimetric viability assay for ex vivo–treated TR-AMs.

Primary TR-AMs isolated from the BALF of naive WT mice and BALF TR-AMs from control patients who underwent routine bronchoscopy for diagnostic purposes and were found to have normal BALF cellularity were treated with 0.1 mL TR-AM medium containing 10% iBALF or rTNFSF14 for 24 hours. For caspase inhibition experiments, cells were preincubated in 50 μM of a specific caspase 3 (Z-DEVD-FMK, catalog FMK004, R&D Systems) or caspase 8 (Z-IETD-FMK, catalog FMK007, R&D Systems) inhibitor for 3 hours prior to BALF treatment. Viability was assessed via colorimetric assay (Cell Counting Kit-8, catalog 96992, MilliporeSigma), per the manufacturer′s instructions, and was considered proportional to the measured light absorbance. Absorbance was measured on an iMark microplate reader (Bio-Rad).

### Caspase 3/-7 and caspase 8 activity.

WT, *Tnfrsf14^–/–^*, and *Ltbr^–/–^* BALF TR-AMs were treated with 0.1 mL TR-AM medium containing 10% day 0 or day 7 iBALF from WT or *Tnfsf14^–/–^* mice for 24 hours. Lyophilized Caspase Glo 3/7 or Caspase Glo 8 substrate (Promega) was resuspended in 10 mL luciferase-containing Caspase Glo buffer (Promega) and added at 0.1 mL/well to the cells. After a 1-hour incubation at room temperature (RT), cells were transferred onto a black 96-well plate for luminescence detection using a 520/25 filter in an FL×800 fluorescence reader (BioTek Instruments). For ligand-blocking experiments, iBALF had been previously incubated with 1 μg/mL mouse anti–mouse TNFSF14 antibody or an isotype control for 1 hour at 4°C prior to iBALF treatment. For TNFSF14 receptor blocking experiments, TR-AMs were pretreated with 1 μg/mL for 1 hour at 37°C, CO_2_, prior to iBALF treatment, to achieve receptor saturation. Antibodies included Armenian hamster anti–mouse TNFRSF14 (CD270 (HVEM) monoclonal antibody, LH1, functional grade, catalog 16–5962-85, eBioscience), Armenian hamster anti–mouse isotype control (catalog 16–4888-85, eBioscience), rat anti–mouse LTβR (clone 4H8-WH2, developed at the laboratory of Carl F. Ware, marketed by AdipoGen Life Sciences, and provided by José Ignacio Rodríguez Barbosa and Maria-Luisa del Rio, University of León, León, Spain), and rat anti–mouse IgG2a isotype control (catalog AG-35B-0002-C050, AdipoGen). To dissect the roles of soluble and transmembrane TNFSF14 on TR-AM apoptosis, flow-sorted day 3 TR-AMs were either treated with iBALF or cocultured with neutrophils at a 1:5 ratio for 24 hours. Marimastat (catalog M2699, MilliporeSigma) was added at a concentration of 10 μM to prevent TNFSF14 shedding.

### S.

*pneumoniae load after* in vivo *infection*. Two days after IAV infection and 6–72 hours after *S. pneumoniae* infection, BALF, lungs, and spleens from coinfected mice were harvested and homogenized. A series of inoculum dilutions in NaCl was prepared for each sample in 1:10 dilution steps. For each dilution step, 4 × 0.01 mL inoculum was pipetted onto a blood agar plate and stored at 37°C overnight. Bacterial load was calculated by counting the average number of separately grown colonies, multiplied by 10^number^ ^of^ ^dilution^ ^step × 100^ (= number of colonies in 1 mL).

### Ex vivo phagocytosis and killing assay.

WT and *Ltbr^–/–^* naive BALF TR-AMs were isolated as previously described. Cells were seeded at 100,000 cells/well on a 96-well, round-bottomed plate and ex vivo infected with *S. pneumoniae* at a MOI of 1,000 for 10 minutes at 37°C. Cells were vigorously washed 5 times in ice-cold PBS to remove any extracellular bacteria and then lysed in water. An inoculum dilution series of cell lysates was pipetted onto blood agar plates as described above. The total colony count on the following day included phagocytosed bacteria. For a flow cytometry–based comparison of day 8 TR-AMs and BMDM *S. pneumoniae* uptake and killing, 100,000 cells of whole BALF cell samples were ex vivo infected with *S. pneumoniae* at a MOI 100 for 10 minutes (*t0*). Cells were washed 3 times with ice-cold PBS and were either fixed and permeabilized using the eBioscience Foxp3/Transcription Factor Staining Buffer Set (catalog 00–5523-00, Invitrogen, Thermo Fisher Scientific), as per the manufacturer′s instructions, or returned to the incubator for an additional 30 minutes (*t1*) in sterile medium, after which the same procedure was performed. Staining was performed in 2 steps, starting with an anti–*S. pneumoniae* antibody (rabbit, catalog PA17259, Invitrogen, Thermo Fisher Scientific) or a rabbit IgG isotype control (catalog ab172730, Abcam) at a concentration of 40 μg/mL for 1 hour at RT, followed by leukocyte surface staining containing a secondary donkey anti–rabbit IgG (H+L) Alexa Fluor 555 antibody (catalog A-31572, Invitrogen). Noninfected samples were used as autofluorescence controls. The phagocytosis capacity was reflected in the percentage of *S. pneumoniae*^+^ cells at *t0*, and the killing capacity at *t1* was determined for each macrophage population as follows: percent killing = 100 − [(% *S. pneumoniae*^+^ cells at *t1*/% *S. pneumoniae*^+^ cells at *t0*) × 100].

### RT^2^ Profiler PCR Arrays.

RT^2^ profiler PCR arrays (Qiagen) were used for pathway or group gene expression analysis of flow-sorted TR-AMs. RNA isolation, genomic DNA elimination, reverse transcription, cDNA synthesis, and qPCR were performed according to the manufacturer′s instructions. In samples with a low RNA amount (<1 μg), a preamplification of cDNA targets preceded qPCR by addition of a PCR master mix (RT^2^ PreAMP PCR Mastermix, Qiagen) and a species- and pathway-specific primer mix (PBM-063Z-RT^2^ PreAMP cDNA Synthesis Primer Mix for Mouse TNF Ligands and Receptors, PBM-212Z – RT² PreAMP cDNA Synthesis Primer Mix for Mouse Cell Death PathwayFinder, catalog 330241, Qiagen) to the cDNA samples. PCR components were added to the 96-well plate format provided by the company (PAMM-063ZC-24-RT² Profiler PCR Array Mouse TNF Signaling Pathway, PAMM-212ZC-12-RT² Profiler PCR Array Mouse Cell Death PathwayFinder, catalog 330231, Qiagen). Real-time PCR was performed using the StepOnePlus Real-Time PCR System and the QuantStudio 3 Real-Time PCR System (Applied Biosystems). Data analysis was performed with the company′s web-based data analysis software (GeneGlobe Data Analysis Center, Qiagen). A detailed description of the data statistical interpretation can be found in the Supplemental Methods.

### scRNA-Seq.

To identify the main leukocyte TNFSF14 source, 500,000 live leukocytes (gated as Sytox^–^CD45^+^) from the homogenized single-cell lung suspensions of IAV-infected mice on days 3 and 7 p.i. were sorted into 0.35 μL 0.04% BSA/PBS^–/–^. Following viability control, 10,000 cells were loaded onto Chromium Chip B (10X Genomics). Gel beads in emulsion (GEM) generation, cDNA synthesis and amplification, and library preparation were performed with the Chromium Single Cell 3′ Reagent Kit, version 3.1 (10X Genomics) following the manufacturer’s protocol. Indexed libraries were sequenced on an Illumina NextSeq 2000. Prior to analysis, reads were aligned against the mouse genome (GRCm38.p6) and quantified using StarSolo (https://github.com/alexdobin/STAR). Analysis was conducted with Scanpy software (https://github.com/theislab/scanpy). After quality filtering, raw cell counts were normalized to the median count over all cells and transformed into log space for variance stabilization. Principal component analysis (PCA) identified 14 and 11 components on days 3 and 7 p.i., respectively. Uniform manifold approximation and projection (UMAP) embedding was created to identify cell type clusters through Leiden clustering. Doublet analysis was conducted on day 7 using Scrublet (https://github.com/swolock/scrublet), leading to the removal of a doublet cluster (118 cells).

### Statistics.

Data are shown in scatterplots as single data points. Means ± SEM per group are indicated by bars and error bars. Statistical significance between 2 groups was calculated using a 2-tailed Student’s *t* test. For comparison between more than 2 groups, significance was determined by 1-way ANOVA, 2-way ANOVA with Tukey’s post hoc test, or Kruskal-Wallis test followed by Dunn′s post hoc comparison test. Survival curves were compared by log-rank (Mantel-Cox) test. A *P* value of less than 0.05 was considered significant. Graphs were prepared using GraphPad Prism 10.2.3 (GraphPad Software).

### Study approval.

Animal experiments were approved by the regional authorities of the State of Hesse (Regierungspraesidium Giessen, Germany) and by the Institutional Ethics Committee at the IBioBA Institute (Buenos Aires, Argentina). Use of human BALF samples was approved by the University of Giessen Ethics Committee, and samples were provided by the biobank of the German Center for Lung Research (DZL). Written informed consent was obtained prior to sample use.

### Data availability.

Data supporting the findings of this study are available within the article and its supplemental material. Values for all data points in graphs are reported in the Supporting Data Values file. ScRNA-Seq data can be accessed under the Gene Expression Omnibus (GEO) database (GEO GSE273805; https://www.ncbi.nlm.nih.gov/geo/query/acc.cgi?acc=GSE273805).

## Author contributions

CM, CP, MRF, KF, KK, CYW, J Better, ME designed and performed experiments and evaluated and interpreted data. CM wrote the manuscript. AIVA and SH designed, performed, and interpreted the scRNA-Seq experiments. IA performed imaging analysis. HS, SG, ML performed sequencing and bioinformatics analyses. J Busche and ADG performed immunohistochemical analysis. KP provided genetically modified mice. MLDR and JIRB provided the neutralizing anti-TNFSF14 and anti-LTβR antibodies. IV performed bronchoscopy for the acquisition of human BALF samples. J Better designed and performed experiments and evaluated and interpreted data. J Bushe performed immunohistochemical analysis together with ADG. CP, SG, ML, ADG, IV, UM, and SH revised the manuscript. SH conceived the scientific question, designed experiments, interpreted data, and financed the study.

## Funding support

- German Research Foundation (Deutsche Forschungsgemeinschaft, DFG) (KFO309 project P2/P9, project no. 284237345; SFB-TR84 project no. 114933180; SFB1021 project no. 197785619).
- Cardiopulmonary Institute (CPI) (DFG EXC 2026 project no. 390649896).
- State of Hesse: LOEWE grant ′diffusible signals (project no. LOEWE/2/13/519/03/06.001[0002]/74, LOEWE professorship, funding line 4a, project ID III L7–519/05.00.002).

## Supplementary Material

Supplemental data

Supplemental data set 1

Supplemental data set 2

Supplemental data set 3

Unedited blot and gel images

Supporting data values

## Version 1 11/18/2025

In-Press Preview

## Version 2 01/16/2026

Electronic publication
