# Supplementary material for: TNF superfamily member 14 drives post-influenza depletion of alveolar macrophages, enabling secondary pneumococcal pneumonia
Source: J Clin Invest. 2025 Nov 18;136(2):e185390. doi: 10.1172/JCI185390 (PMC12807475; doi:10.1172/JCI185390)

SDS-PAGE

Western blot uncropped

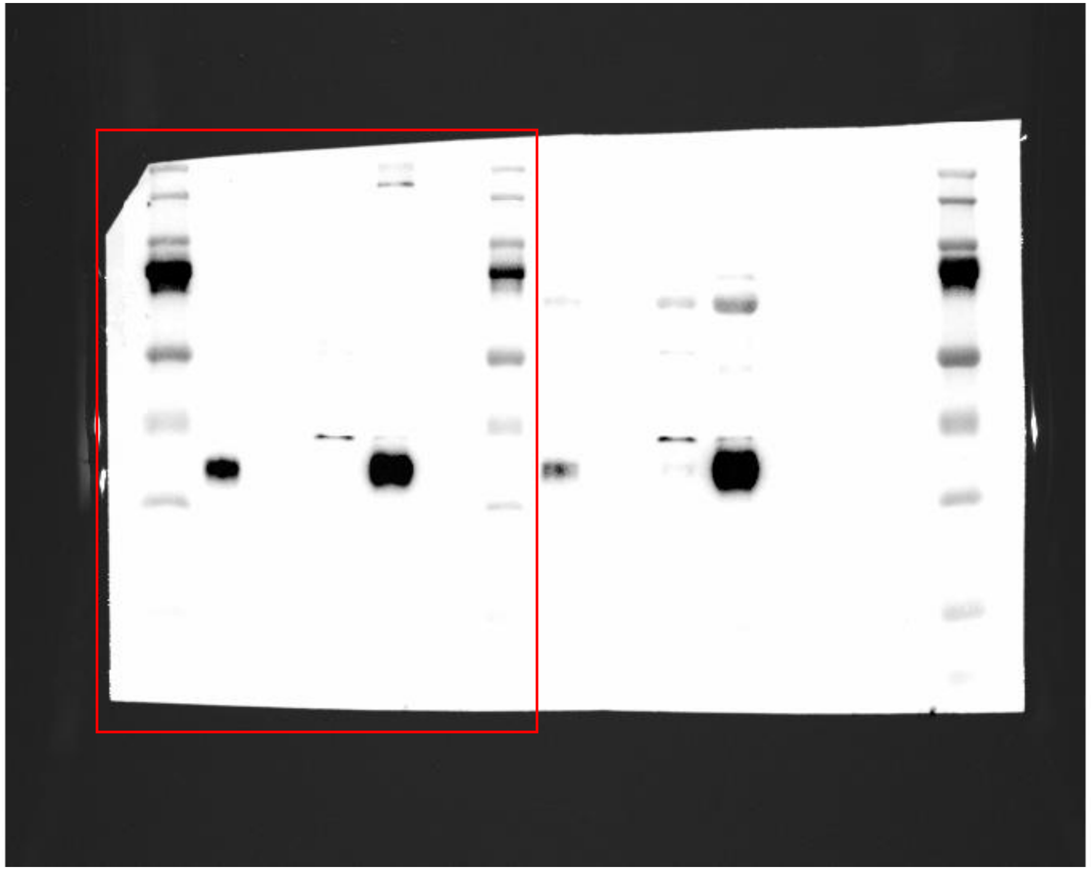

Reducing denaturated

Non-reducing denaturated

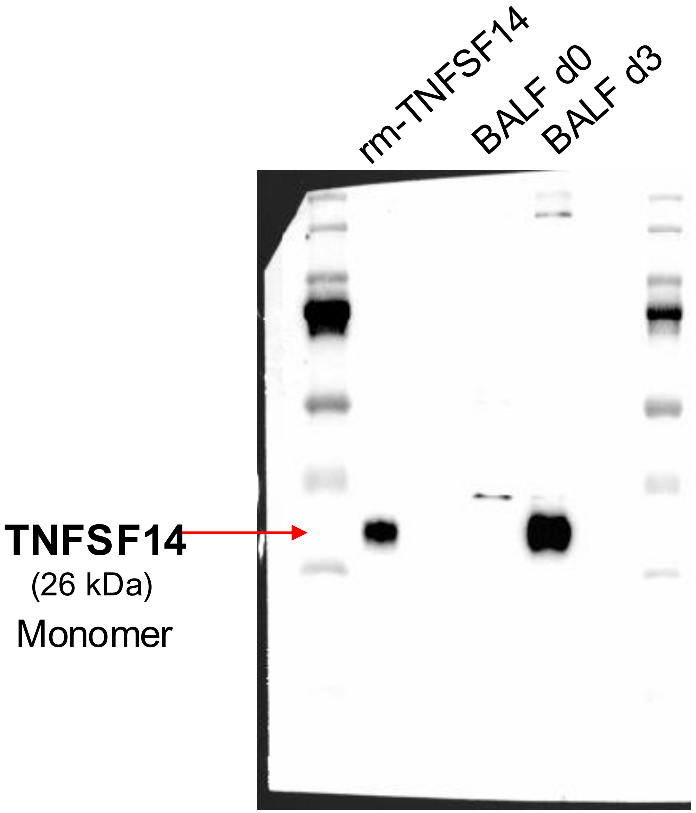

Blue Native PAGE

Western blot (BN-PAGE) uncropped

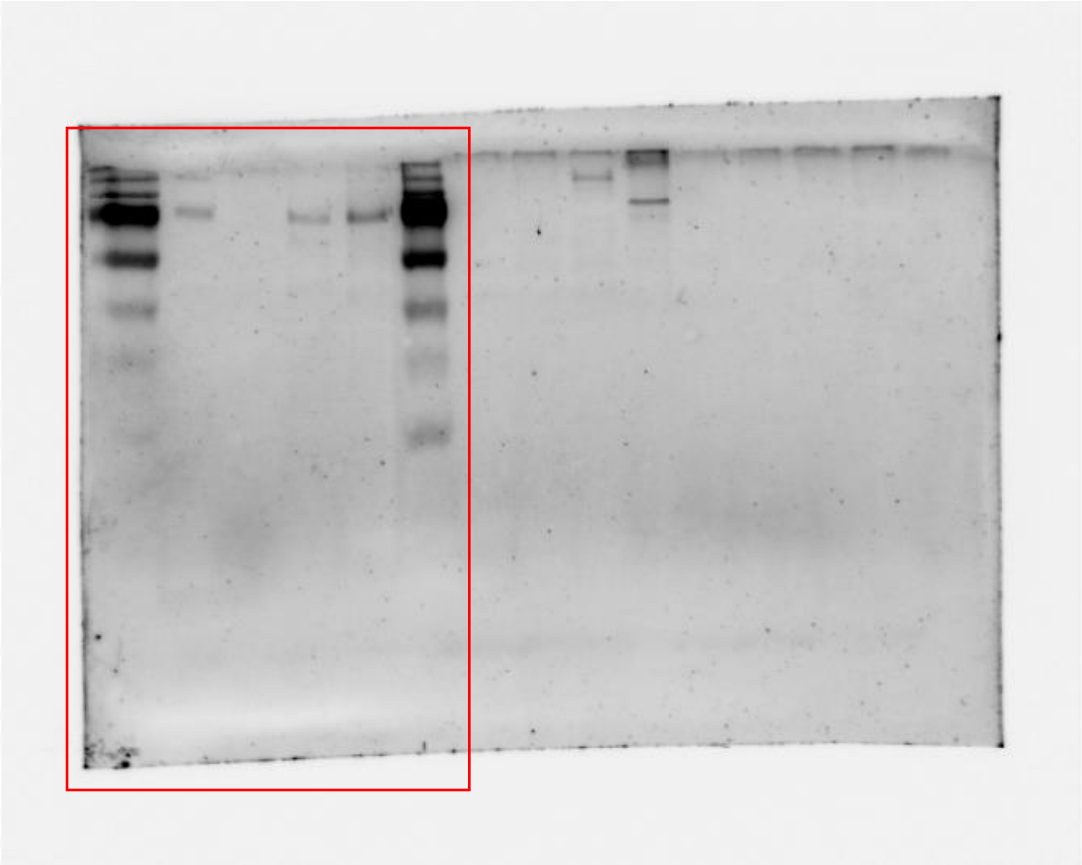

+native sample buffer
(#161-0738, Bio-Rad)

+coomassie blue G250
(0.1% w/v)

+native sample buffer
(#161-0738, Bio-Rad)

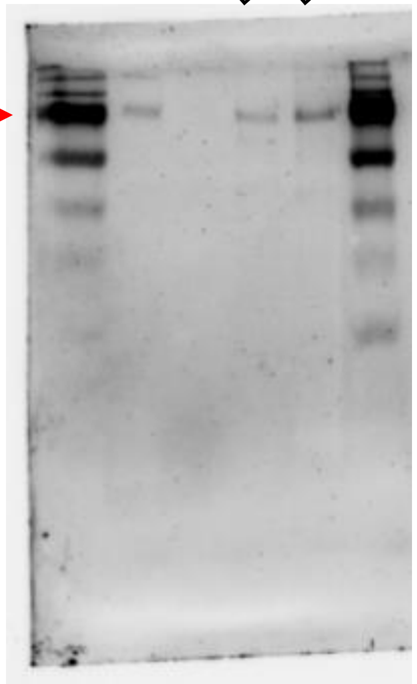

Supplement: Unedited blot and gel images [file jci-136-185390-s181.pdf]
