# Supplementary material for: TNF superfamily member 14 drives post-influenza depletion of alveolar macrophages, enabling secondary pneumococcal pneumonia
Source: J Clin Invest. 2025 Nov 18;136(2):e185390. doi: 10.1172/JCI185390 (PMC12807475; doi:10.1172/JCI185390)
Supplement: Supplemental data [file jci-136-185390-s177.pdf]

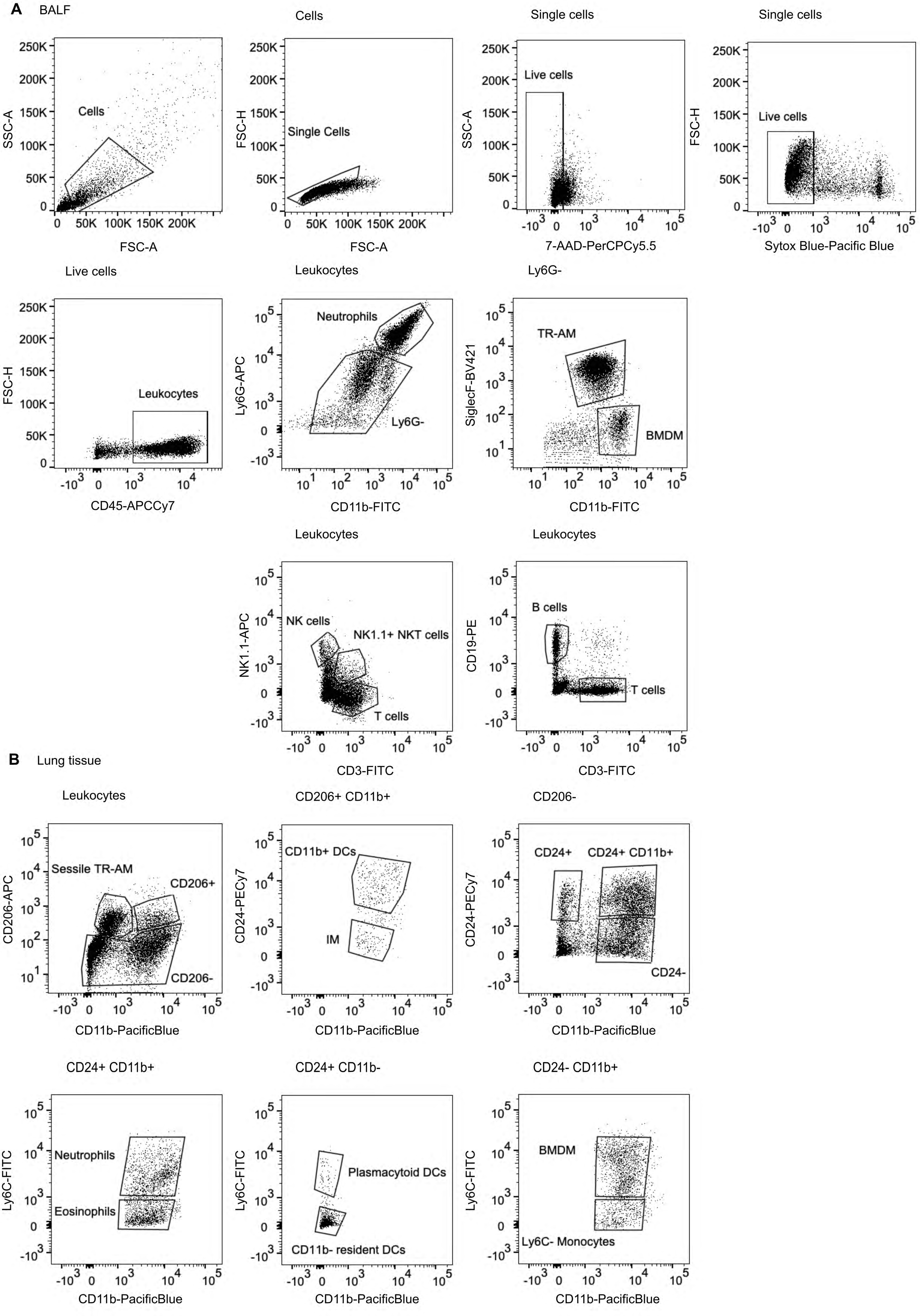
A
BALF
Cells
SSC-A
FSC-A
Single cells
Single Cells
FSC-H
Live cells
7-AAD-PerCPCy5.5
Sytox Blue-Pacific Blue
Leukocytes
CD45-APCCy7
Neutrophils
Ly6G-
Ly6G-APC
CD11b-FITC
TR-AM
BMDM
SiglecF-BV421
NK cells
NK1.1+ NKT cells
T cells
NK1.1-APC
CD3-FITC
B cells
CD19-PE
B
Lung tissue
Sessile TR-AM
CD206+
CD206-
CD206-APC
CD11b-PacificBlue
CD206+ CD11b+
CD11b+ DCs
IM
CD24-PECy7
CD24+
CD24+ CD11b+
CD24-
CD24+ CD11b-
CD24- CD11b+
Eosinophils
Ly6C-FITC
Plasmacytoid DCs
CD11b- resident DCs
Ly6C- Monocytes

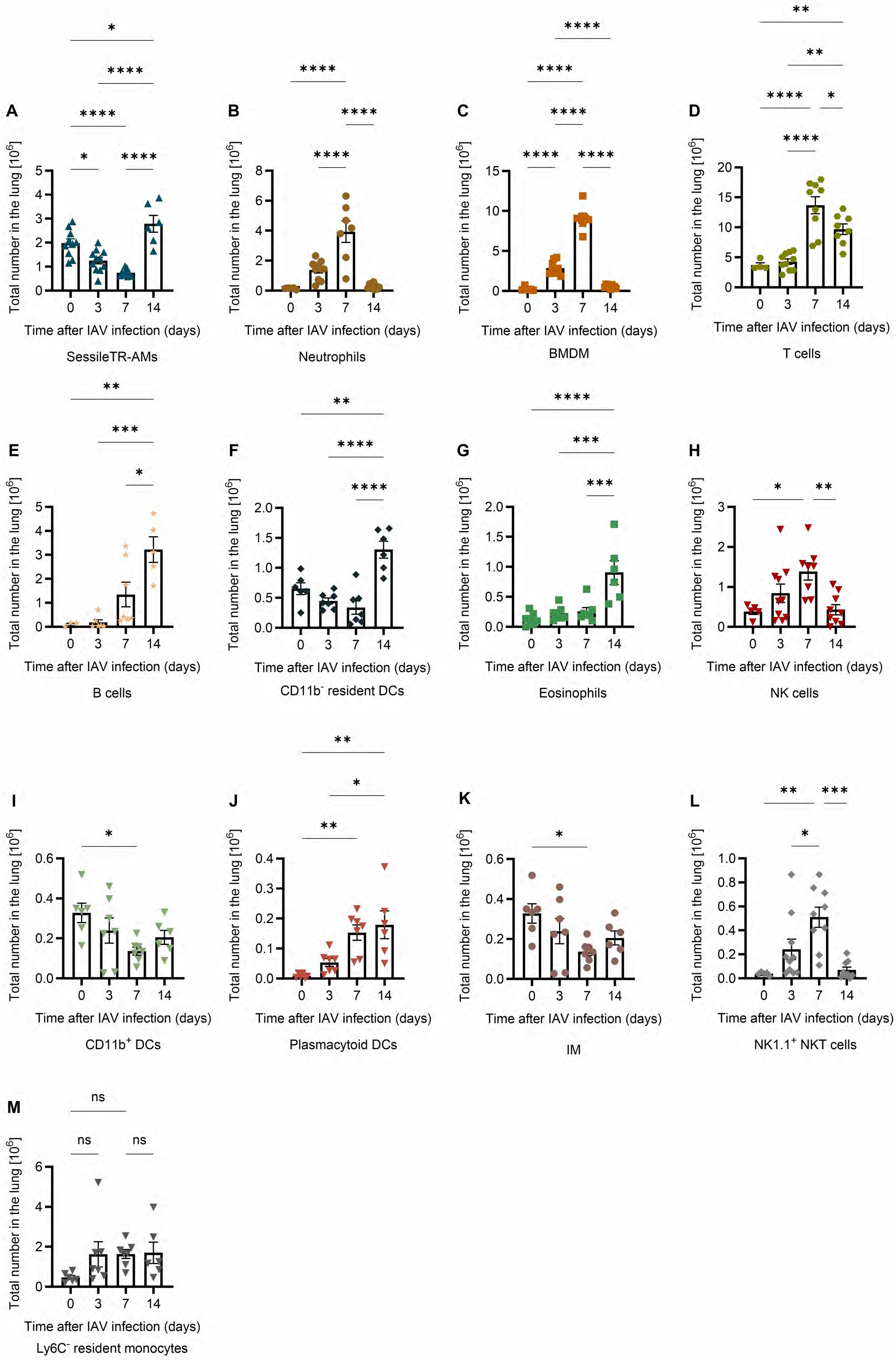
A
B
C
D
E
F
G
H
I
J
K
L
M
Total number in the lung [10^6]
Time after IAV infection (days)
0
3
7
14
*
**
***
****
ns
SessileTR-AMs
Neutrophils
BMDM
T cells
B cells
CD11b⁻ resident DCs
Eosinophils
NK cells
CD11b⁺ DCs
Plasmacytoid DCs
IM
NK1.1⁺ NKT cells
Ly6C⁻ resident monocytes

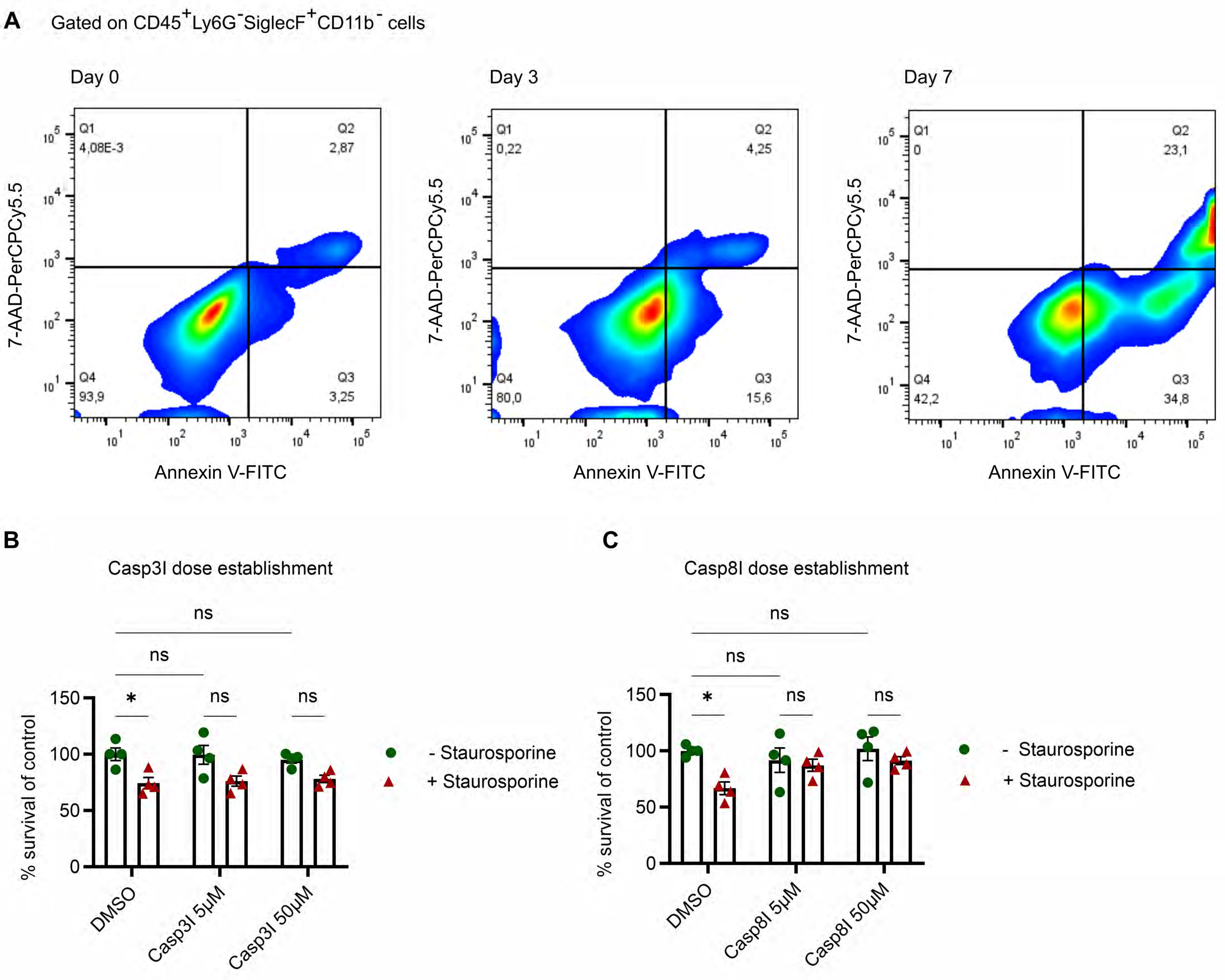
A
Gated on CD45+Ly6G-SiglecF+CD11b- cells
Day 0
Day 3
Day 7
7-AAD-PerCPCy5.5
Annexin V-FITC
Q1
4,08E-3
Q2
2,87
Q4
93,9
Q3
3,25
Q1
0,22
Q2
4,25
Q4
80,0
Q3
15,6
Q1
0
Q2
23,1
Q4
42,2
Q3
34,8
B
Casp3I dose establishment
ns
ns
*
ns
ns
% survival of control
DMSO
Casp3I 5µM
Casp3I 50µM
- Staurosporine
+ Staurosporine
C
Casp8I dose establishment
ns
ns
*
ns
ns
% survival of control
DMSO
Casp8I 5µM
Casp8I 50µM
- Staurosporine
+ Staurosporine

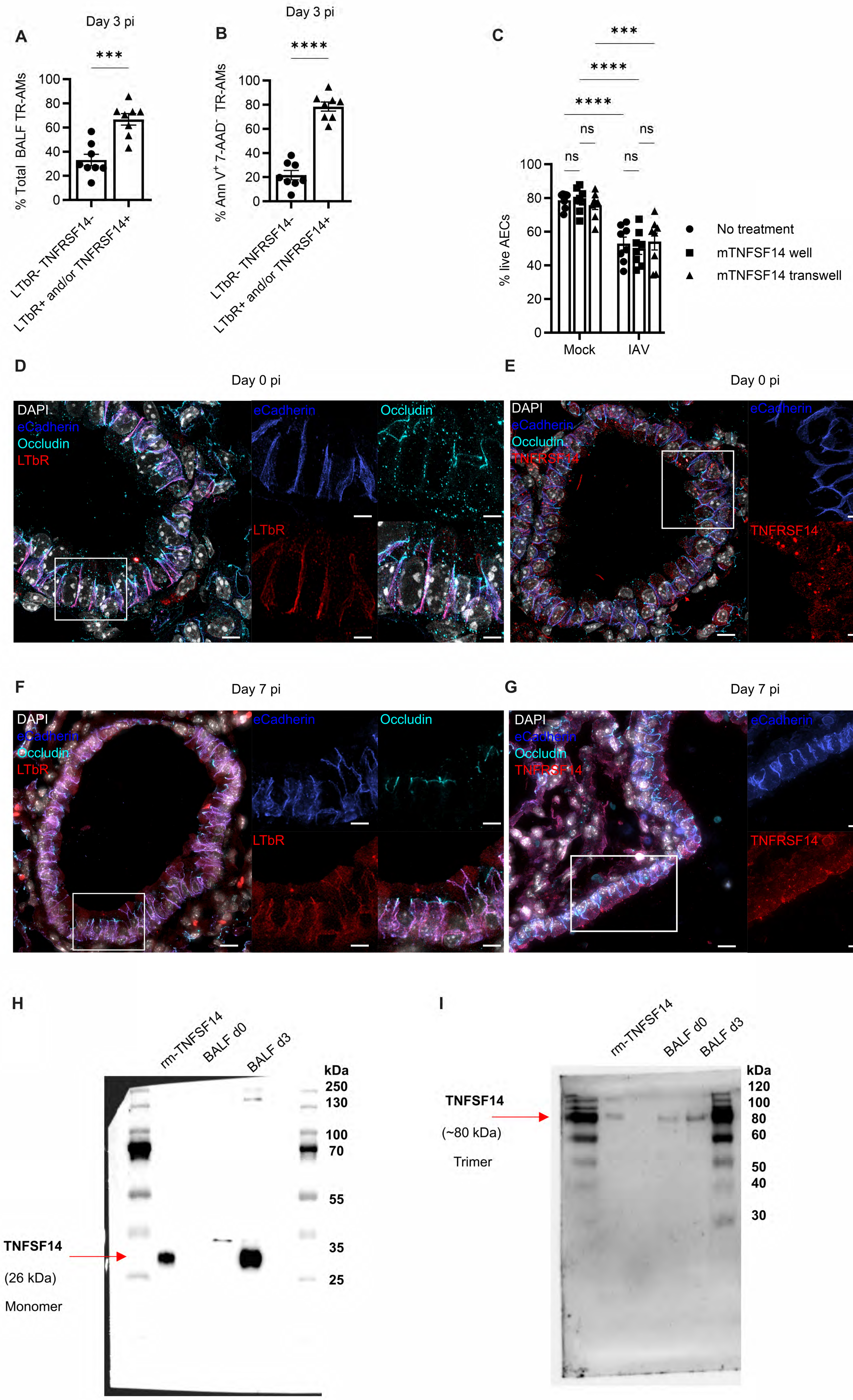
A
Day 3 pi
***
% Total BALF TR-AMs
100
80
60
40
20
0
LTbR- TNFRSF14-
LTbR+ and/or TNFRSF14+
B
Day 3 pi
****
% Ann V+ 7-AAD- TR-AMs
LTbR- TNFRSF14-
LTbR+ and/or TNFRSF14+
C
***
****
****
ns
ns
ns
ns
% live AECs
Mock
IAV
No treatment
mTNFSF14 well
mTNFSF14 transwell
D
Day 0 pi
DAPI
eCadherin
Occludin
LTbR
E
Day 0 pi
TNFRSF14
F
Day 7 pi
G
Day 7 pi
H
rm-TNFSF14
BALF d0
BALF d3
kDa
250
130
100
70
55
35
25
TNFSF14
(26 kDa)
Monomer
I
rm-TNFSF14
BALF d0
BALF d3
kDa
120
100
80
60
50
40
30
TNFSF14
(~80 kDa)
Trimer

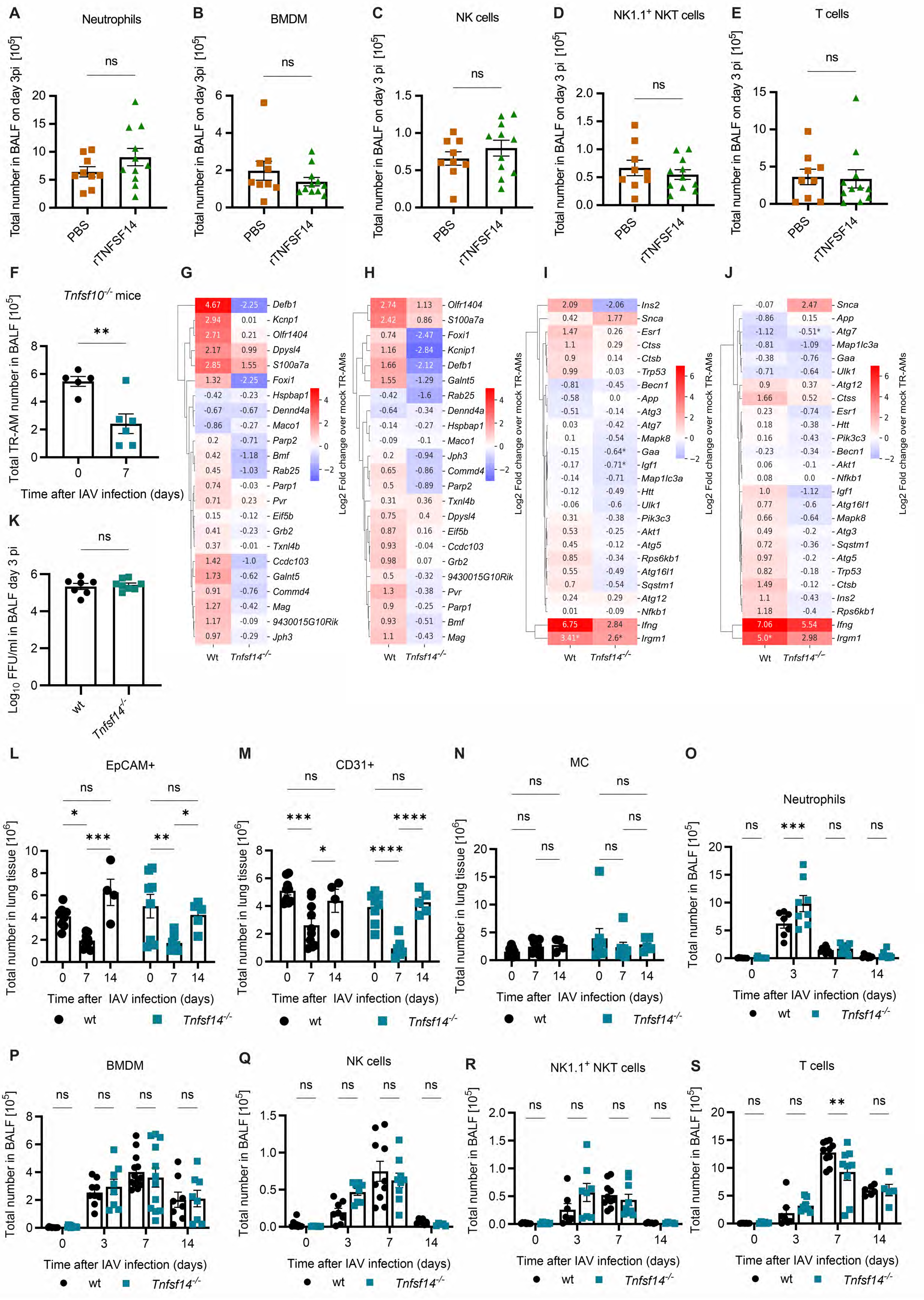
A
Neutrophils
Total number in BALF on day 3pi [10^5]
ns
PBS
rTNFSF14
B
BMDM
Total number in BALF on day 3pi [10^5]
ns
C
NK cells
Total number in BALF on day 3 pi [10^5]
ns
D
NK1.1+ NKT cells
Total number in BALF on day 3 pi [10^5]
ns
E
T cells
Total number in BALF on day 3 pi [10^5]
ns
F
Tnfsf10-/- mice
Total TR-AM number in BALF [10^5]
**
Time after IAV infection (days)
G
4.67 -2.25 Defb1
2.94 0.01 Kcnp1
2.71 0.21 Olfr1404
2.17 0.99 Dpysl4
2.85 1.55 S100a7a
1.32 -2.25 Foxi1
-0.42 -0.23 Hspbap1
-0.67 -0.67 Dennd4a
-0.86 -0.27 Maco1
0.2 -0.71 Parp2
0.42 -1.18 Bmf
0.45 -1.03 Rab25
0.74 -0.03 Parp1
0.71 0.23 Pvr
0.15 -0.12 Eif5b
0.41 -0.23 Grb2
0.37 -0.01 Txnl4b
1.42 -1.0 Ccdc103
1.73 -0.62 Galnt5
0.91 -0.76 Commd4
1.27 -0.42 Mag
1.17 -0.09 9430015G10Rik
0.97 -0.29 Jph3
Wt
Tnfsf14-/-
Log2 Fold change over mock TR-AMs
H
2.74 1.13 Olfr1404
2.42 0.86 S100a7a
0.74 -2.47 Foxi1
1.16 -2.84 Kcnip1
1.66 -2.12 Defb1
1.55 -1.29 Galnt5
-0.42 -1.6 Rab25
-0.64 -0.34 Dennd4a
-0.14 -0.27 Hspbap1
-0.09 -0.1 Maco1
0.2 -0.94 Jph3
0.65 -0.86 Commd4
0.5 -0.89 Parp2
0.31 0.36 Txnl4b
0.75 0.4 Dpysl4
0.87 0.16 Eif5b
0.93 -0.04 Ccdc103
0.98 0.07 Grb2
0.5 -0.32 9430015G10Rik
1.3 -0.38 Pvr
0.9 -0.25 Parp1
0.93 -0.51 Bmf
1.1 -0.43 Mag
Wt
Tnfsf14-/-
Log2 Fold change over mock TR-AMs
I
2.09 -2.06 Ins2
0.42 1.77 Snca
1.47 0.26 Esr1
1.1 0.29 Ctss
0.9 0.14 Ctsb
0.99 -0.03 Trp53
-0.81 -0.45 Becn1
-0.58 0.0 App
-0.51 -0.14 Atg3
0.03 -0.42 Atg7
0.1 -0.54 Mapk8
-0.15 -0.64* Gaa
-0.17 -0.71* Igf1
-0.14 -0.71 Map1lc3a
-0.12 -0.49 Htt
-0.06 -0.6 Ulk1
0.31 -0.38 Pik3c3
0.53 -0.25 Akt1
0.45 -0.12 Atg5
0.85 -0.34 Rps6kb1
0.55 -0.49 Atg16l1
0.7 -0.54 Sqstm1
0.24 0.29 Atg12
0.01 -0.09 Nfkb1
6.75 2.84 Ifng
3.41* 2.6* Irgm1
Wt
Tnfsf14-/-
Log2 Fold change over mock TR-AMs
J
-0.07 2.47 Snca
-0.86 0.15 App
-1.12 -0.51* Atg7
-0.81 -1.09 Map1lc3a
-0.38 -0.76 Gaa
-0.71 -0.64 Ulk1
0.9 0.37 Atg12
1.66 0.52 Ctss
0.23 -0.74 Esr1
0.18 -0.38 Htt
0.16 -0.43 Pik3c3
-0.23 -0.34 Becn1
0.06 -0.1 Akt1
0.08 0.0 Nfkb1
1.0 -1.12 Igf1
0.77 -0.6 Atg16l1
0.66 -0.64 Mapk8
0.49 -0.2 Atg3
0.72 -0.36 Sqstm1
0.97 -0.2 Atg5
0.82 -0.18 Trp53
1.49 -0.12 Ctsb
1.1 -0.43 Ins2
1.18 -0.4 Rps6kb1
7.06 5.54 Ifng
5.0* 2.98 Irgm1
Wt
Tnfsf14-/-
Log2 Fold change over mock TR-AMs
K
Log10 FFU/ml in BALF day 3 pi
ns
wt
Tnfsf14-/-
L
EpCAM+
Total number in lung tissue [10^6]
ns
*
***
**
Time after IAV infection (days)
wt
Tnfsf14-/-
M
CD31+
Total number in lung tissue [10^6]
ns
***
****
*
Time after IAV infection (days)
N
MC
Total number in lung tissue [10^6]
ns
Time after IAV infection (days)
O
Neutrophils
Total number in BALF [10^5]
ns
***
Time after IAV infection (days)
P
BMDM
Total number in BALF [10^5]
ns
Time after IAV infection (days)
Q
NK cells
Total number in BALF [10^5]
ns
Time after IAV infection (days)
R
NK1.1+ NKT cells
Total number in BALF [10^5]
ns
Time after IAV infection (days)
S
T cells
Total number in BALF [10^5]
ns
**
Time after IAV infection (days)
wt
Tnfsf14-/-

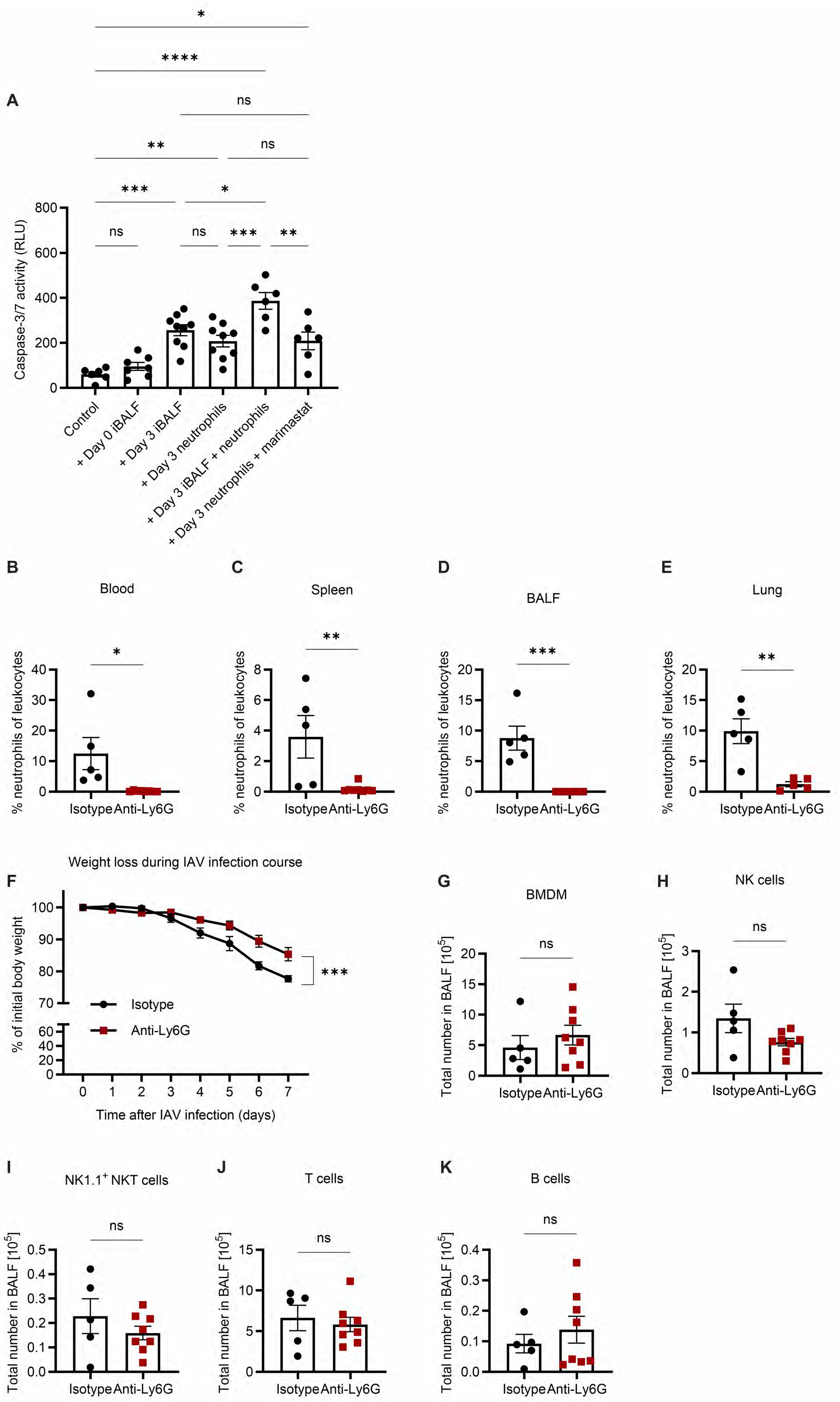
A
Caspase-3/7 activity (RLU)
Control
+ Day 0 iBALF
+ Day 3 iBALF
+ Day 3 neutrophils
+ Day 3 iBALF + neutrophils
+ Day 3 neutrophils + marimastat
B
Blood
C
Spleen
D
BALF
E
Lung
% neutrophils of leukocytes
Isotype
Anti-Ly6G
F
Weight loss during IAV infection course
% of initial body weight
Time after IAV infection (days)
G
BMDM
H
NK cells
I
NK1.1+ NKT cells
J
T cells
K
B cells
Total number in BALF [10^5]

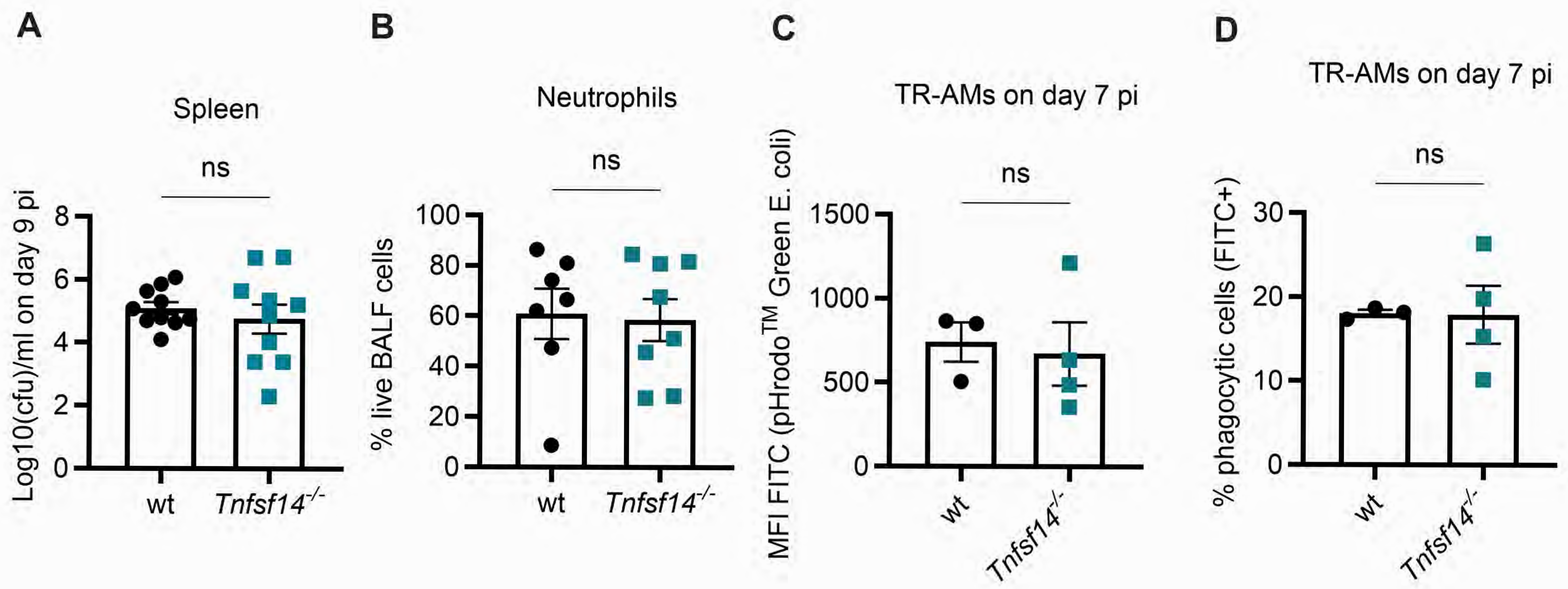
A
Spleen
ns
Log10(cfu)/ml on day 9 pi
8
6
4
2
0
wt
Tnfsf14-/-
B
Neutrophils
ns
% live BALF cells
100
80
60
40
20
0
wt
Tnfsf14-/-
C
TR-AMs on day 7 pi
ns
MFI FITC (pHrodoTM Green E. coli)
1500
1000
500
0
wt
Tnfsf14-/-
D
TR-AMs on day 7 pi
ns
% phagocytic cells (FITC+)
30
20
10
0
wt
Tnfsf14-/-

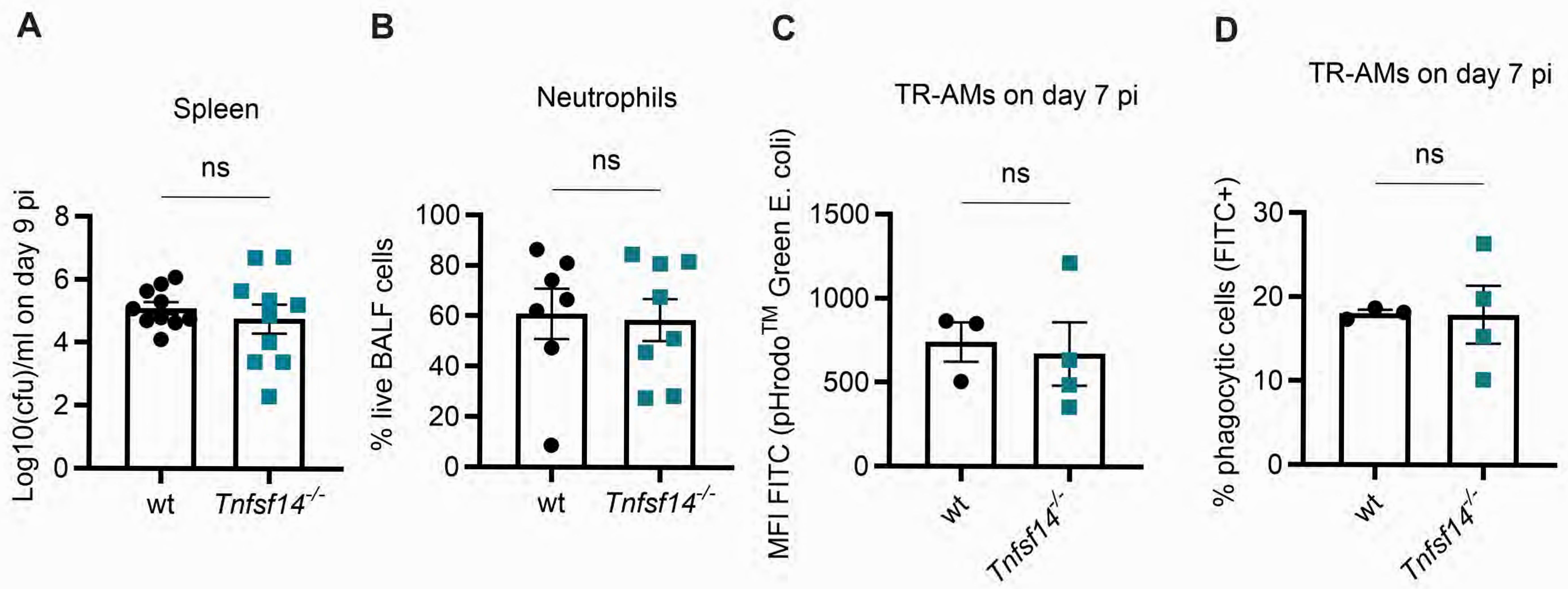
A
Spleen
ns
Log10(cfu)/ml on day 9 pi
0
2
4
6
8
wt
Tnfsf14-/-
B
Neutrophils
ns
% live BALF cells
0
20
40
60
80
100
wt
Tnfsf14-/-
C
TR-AMs on day 7 pi
ns
MFI FITC (pHrodoTM Green E. coli)
0
500
1000
1500
wt
Tnfsf14-/-
D
TR-AMs on day 7 pi
ns
% phagocytic cells (FITC+)
0
10
20
30
wt
Tnfsf14-/-

Supplemental Figure 1. Gating strategy for the identification of different immune cell populations in murine BALF (A) and lung tissue (B) per flow cytometry analysis. Representative plots following multicolor staining of BALF and lung-tissue immune cells, as described in the ´Supplemental Methods´ document. Gating strategies were set according to the appropriate isotype and fluorescence minus one (FMO) controls.

Supplemental Figure 2. Immune cell kinetics in the lung tissue of IAV-infected mice over the infection course. Following BALF extraction, lungs of wild-type mice infected with 500ffu IAV were harvested at different time points. Immune cell populations were identified per flow cytometry analysis, including sessile TR-AMs (n=6-11) (**A**), neutrophils (n=5-10) (**B**), BMDM (n=5-10) (**C**), T cells (n=4-10) (**D**), B cells (n=3-7) (**E**), CD11b^{-} resident DCs, (n=6-7) (**F**), eosinophils (n=6-7) (**G**), NK cells (n=5-10) (**H**), CD11b^{+} DCs (n=6-7) (**I**), pDCs (n=6-7) (**J**), IM (n=6-7) (**K**), NK1.1^{+} NKT cells (n=5-10) (**L**), and Ly6C^{-} resident monocytes (n=6-7) (**M**). Data shown pooled from nine independent experiments, mean ± SEM is depicted. Significance was determined by 1-way ANOVA with Tukey's posthoc test; *p<0.05, **p<0.01, ***p <0.001, ****p <0.0001. (p)DCs: (plasmacytoid) dendritic cells, IM: interstitial macrophages, NK: natural killer.

Supplemental Figure 3. TR-AM apoptosis can be attenuated through the use of a caspase inhibitor. (**A**) Gating strategy for the quantification of apoptotic TR-AMs in the BALF of IAV-infected mice on days 0, 3, and 7 pi. (**B-C**) TR-AM viability following treatment with 0.5μM staurosporine and different doses of a caspase-3 (**B**) or a caspase-8 (**C**) inhibitor, values depicted as % survival of control. DMSO-treated cells set at 100%. Graphs represent means ± SEM, n=4 per condition. Data pooled from three independent experiments. Significance was determined by 2-way ANOVA with Tukey's posthoc test; *$p<0.05$.

Supplemental Figure 4. TNFSF14 orchestrates post-influenza TR-AM apoptosis in a cell-specific manner. (**A-B**) TNFSF14 receptor- positive cells as percentage of all BALF TR-AMs on day 3 pi (**A**) and percentage of apoptotic TR-AMs based on TNFSF14 receptor expression at the same time point (**B**). Graphs represent means ± SEM, n=8 per group, data poled from two independent experiments. (**C**) Percentage of live transwell-seeded AEC after 24h IAV infection and treatment with rTNFSF14. Graphs represent means ± SEM, n=9 per group, data pooled from two independent experiments. (**D-G**) Immunofluorescence analysis revealing a basolateral LTbR expression (**D,F**) and a non-preferential, cytoplasmic TNFRSF14 expression (**E,G**) on epithelial cells on days 0 (**D-E**) and 7 pi (**F-G**). Scale bar for smaller panels set at 5μm, for larger panels set at 10μm (**D-E**) or 20μm (**F-G**). Data representative of three independent experiments. (**H-I**) SDS-PAGE (**H**) and blue native PAGE (**I**) for TNFSF14 in naïve and day 3 BALF samples with mouse rTNFSF14 (rmTNFSF14) as a positive control, demonstrating a single band at 26kDa upon denaturation. Under naïve conditions TNFSF14 appeared around 80kDa, reflecting the presence of the homotrimer in the BALF. Data representative of three independent experiments. Graphs represent means ± SEM, n=6-8. Data pooled from three independent experiments. Significance was determined by unpaired 2-tailed t-test and two-way ANOVA; ***p <0.001, ****p <0.0001.

Supplemental Figure 5. Lack of TNFSF14 attenuates TR-AM death without heavily affecting other cell population kinetics. (**A-E**) BALF neutrophils (**A**), BMDM (**B**), NK cells (**C**), NK1.1$^+$ NKT cells (**D**), and T cells (**E**) on day 3 pi after rTNFSF14 treatment on days 1 and 2, n=9-11, means ± SEM, data pooled from three independent experiments. (**F**) Quantification of TR-AM numbers in the BALF of IAV-infected *Tnfsf10*$^{-/-}$ mice on day 7 pi (means ± SEM, n=5-6, data pooled from three independent experiments). (**G-J**) Heat maps depicting fold change of necrosis- (**G-H**) and autophagy-related (**I-J**) genes on day 3 pi (**G, I**) and day 7 pi (**H, J**) over mock-infected wt and *Tnfsf14*$^{-/-}$ TR-AMs, n=3-5, data from five independent experiments. Wt data extracted from the data set presented in Figure 2E. (**K**) Viral titers in the BALF of wt and *Tnfsf14*$^{-/-}$ mice on day 3 pi (means ± SEM, n=4, data representative of three independent experiments). (**L-N**) Lung epithelial (EpCAM$^{+,}$ **L**), endothelial (CD31+, **M**), and mesenchymal cells (MC, **N**) of wt and *Tnfsf14*$^{-/-}$ mice after IAV infection (means ± SEM, n=4-10, data pooled from ten independent experiments). (**O-S**) Immune cell populations in the BALF of wt and *Tnfsf14*$^{-/-}$ mice, including neutrophils (**O**), BMDM (**P**), NK cells (**Q**), NK1.1$^+$ NKT cells (**R**), and T cells (**S**). Graphs represent means ± SEM, n=5-13, data pooled from fourteen independent experiments, wt controls including data presented in Figure 1I. Significance was determined by unpaired 2-tailed t-test and 2-way ANOVA with Tukey's posthoc test; *p<0.05, **p<0.01, ***p <0.001, ****p <0.0001.

Supplemental Figure 6. Neutrophil depletion attenuates weight loss without affecting leukocyte influx after IAV infection. (**A**) Caspase-3/7 activation after treatment of day 3 TR-AMs with iBALF or co-culture with neutrophils plus a metalloproteinase inhibitor, n=6-9, data pooled from three independent experiments. (**B-E**) Neutrophils depicted as percentage of total leukocyte numbers after neutrophil depletion, in the blood (**B**), spleen (**C**), BALF (**D**), and lungs (**E**) of IAV-infected mice on day 7 pi (means ± SEM, n=5-8, data pooled from two independent experiments). (**F**) Body weight as percentage of initial weight after IAV infection and neutrophil depletion, n=7-11, data pooled from four independent experiments. (**G-K**) BALF immune populations on day 7 pi after neutrophil depletion, including BMDM, **(G)**, NK cells **(H)**, $NK1.1^+$ NKT cells (**I**), T cells (**J**), and B cells (**K**). Graphs represent means ± SEM, n=5-8, data from two independent experiments. Significance was determined by unpaired 2-tailed t-test for two groups, including the AUC between the two treatment groups for (**F**); *p<0.05, **p<0.01, ***p <0.001, ****p <0.0001.

Supplemental Figure 7. Loss of TNFSF14 does not impact neutrophil influx, spleen bacterial burden, or TR-AM phagocytosis capacity after IAV/Spn co-infection. (**A**) Spn burden in the spleens of wt and *Tnfsf14*$^{-/-}$ mice 9 days after IAV infection and 48h after Spn infection (means ± SEM, n=10, data pooled from eight different experiments). (**B**) Neutrophils depicted as percentage of total live cells in the BALF of wt and *Tnfsf14*$^{-/-}$ mice at the same time point (means ± SEM, n=7-8, data pooled from five independent experiments). (**C-D**) Phagocytosis capacity of wt and *Tnfsf14*$^{-/-}$ day 7 TR-AMs depicted as FITC signal normalized to non-infected TR-AMs (**C**) and percentage of FITC+ TR-AMs (phagocytic cells, **D**), following ex vivo incubation with pHrodo^{TM} Green *Escherichia coli* BioParticles^{TM} (means ± SEM, n=3-4, data representative of three independent experiments). Significance was determined by unpaired 2-tailed t-test.

Methods

Bone marrow-derived neutrophil isolation. For flow cytometry analysis of TNFSF14 expression on bone marrow (BM) neutrophils, femur and tibia were extracted from non-infected mice after isoflurane-induced euthanasia. Any remaining muscles or fur were removed using PBS-soaked gauze, bones were cut with a scalpel on both sides and flushed with 5mL medium (DMEM+10%FCS), to collect the bone marrow. Cells were centrifuged at 300g, 10min, 4°C, washed in 10mL MACS buffer (PBS, 7.4% EDTA, 0.5% FCS pH 7.2), and filtered through a 100µm filter. Following an additional centrifugation step at 300g, 10min, 4°C, cells were resuspended in medium (DMEM +10% FCS+2,5% Hepes+1% L-glutamine/penicillin/streptomycin). Following cell count, neutrophil isolation was performed with a neutrophil isolation kit (cat. 130-097-658, Miltenyi), according to the manufacturer´s instructions. More specifically, cell suspensions were centrifuged at 300g for 10min, resuspended in 0.2mL buffer per 5×10^7 total cells, and incubated in 0.05mL of neutrophil biotin-antibody cocktail for 10min at 4°C. After a washing step, cells were incubated in 0.1mL of anti-biotin microbeads per 5×10^7 cells for 15min at 4°C, washed, recentrifuged, and resuspended in 0.5mL buffer. Magnetic separation was performed by adding the cell suspensions to LS magnetic separation columns (Miltenyi) on a MidiMACS magnetic separator (Miltenyi), with the flow-through containing unlabeled, enriched neutrophils.

Flow cytometry and FACS. Multicolor flow cytometry analysis and FACS were performed on an LSR Fortessa and a BD FACSAria III cell sorter using the FlowJoTM v10.7 software (BD Biosciences). Cells obtained from the BALF and homogenized lung tissue of naïve and infected animals were stained with 7-AAD (cat. 420404, BioLegend) or Sytox™ Blue Dead Cell Stain (cat. S34857, Invitrogen) for dead cell exclusion, or with a FITC Annexin V apoptosis detection kit with 7-AAD (cat. 640922, Biolegend), for apoptosis analysis. Primary antibodies used for flow cytometry and cell

sorting experiments included goat anti-mouse influenza A Virus (1:50, cat. ab20841, abcam), anti-LIGHT/TNFSF14 antibody (rabbit anti-mouse, 1:500, cat. ab305235, abcam), rat anti-mouse CD45 APC/Cy7, PE/Cy7 (1:100, clone 30-F11, BioLegend), rat anti-mouse Ly-6G/Ly-6C (Gr-1) PE/Cy7 (1:100, clone RB6-8C5, BioLegend), rat anti-mouse SiglecF PE, BV421 (1:50, clone E50-2440, BD Biosciences), Armenian hamster anti-mouse CD11c FITC, PE/Cy7 (1:20, clone N418, BioLegend), rat anti-mouse Ly-6G APC (1:50, clone 1A8, BioLegend), rat anti-mouse/human CD11b FITC, Pacific Blue (1:50, clone M1/70, BioLegend), Syrian hamster anti-mouse CD3e FITC (1:50, clone 500A2, BioLegend), rat anti-mouse CD19 PE (1:50, clone 6D5, BioLegend), rat anti-mouse CD206 APC (1:20, clone C068C2, BioLegend), rat anti-mouse CD24 PE/Cy7 (1:200, clone M1/69, BioLegend), mouse anti-mouse CD64 (FcγRI) PE/Cy7 (1:50, clone X54-5/7.1, BioLegend), rat anti-mouse CD326 (Ep-CAM) APC/Cy7 (1:50, clone G8.8, BioLegend), rat anti-mouse CD31 Alexa Fluor^{TM} 488 (1:100, clone 390, BioLegend), and mouse anti-mouse NK1.1 APC (1:50, clone PK136, BD Biosciences). Secondary antibodies included rabbit anti-goat IgG (H+L) cross-adsorbed secondary antibody Alexa Fluor^{TM} 647 (1:100, clone A-21446, Invitrogen), goat anti-rabbit IgG (H+L) cross-adsorbed secondary antibody Alexa Fluor^{TM} 555 (1:100, clone A-21428, Invitrogen), and goat anti-rabbit IgG (H+L) secondary antibody FITC (1:100, clone 31635, Invitrogen). Total cell numbers were determined on a NucleoCounter® NC-3000TM (ChemoMetec). Flow-sorted cells were resuspended in 350µl RLT^{TM} buffer (Qiagen) for further gene expression analyses.

Histology, IHC, and immunofluorescence. Lungs of IAV-, IAV/Spn, and PBS-infected mice were fixed for 24h in 4% paraformaldehyde, embedded in paraffin, and sliced in 3-5 µm thick sections. Hematoxylin and eosin staining was performed to depict leukocyte influx. For IHC, antigen retrieval was performed through protease addition (0,1g protease from Streptomyces griseus, cat. A1852, Pan Reac AppliChem, ITW

Reagents, activity: 10474,48 IU/mg) in 100 mL PBS (10´, 37°C). Blocking serum [PBS, Roti-ImmunoBlock (cat. T144.1, Roth) and goat normal serum] was applied for 30min at RT and the primary antibody (anti-LIGHT/TNFSF14 rabbit anti-mouse, cat. ab203578, abcam) was applied overnight at 4°C. The next day, slides were incubated in the secondary antibody [goat anti-rabbit IgG (H+L), biotinylated, cat. BA-1000, Vector Laboratories)]. Images were obtained at the Olympus BX 41 microscope using the cellSens imaging software (Olympus Corporation ver. 1.18) and were edited with Photoshop CS5 (64 bit). TNFSF14 expression was determined in a semi-quantitative manner, based on the frequency and intensity of positive signal in the lung interstitium, airspaces, subpleural regions, and bronchial epithelium. A quantification system was developed, ranging from 0 - no signal present- to 4, when TNFSF14 signal was vividly present in the examined tissue. For LTbR and TNFRSF14 expression on epithelial cells, deparaffinized lungs from IAV-infected mice on day 0 and 7 pi were stained for occludin (OC-3F10, mouse anti-mouse, 1:100, cat. 33-1511, Invitrogen), TNFRSF14 (rabbit anti-mouse, 5µg/mL, cat. PA5-29780, Invitrogen), LTbR (rabbit anti-mouse, 1:200, cat. PA5-102743, Invitrogen), and e-cadherin (goat anti-mouse, 1:200, cat. AF748-SP, R&D Systems), overnight at 4°C, following antigen retrieval (permeabilization buffer containing 10mM sodium citrate, cat. 005000, Invitrogen, 0.05% Tween, pH 6.0, applied at 100°C for 20min), and blocking for 1h at RT (10% horse serum, 1% BSA in PBS). Secondary antibodies donkey anti-goat Alexa Fluor™ 647 (1:1000, cat. ab150135, abcam), donkey anti-mouse Alexa Fluor™ 488 (1:1000, cat. ab150105, abcam), and donkey anti-rabbit Alexa Fluor™ 555 (1:1000, cat. ab150074, abcam) were applied the next day for 1h at RT, and imaging was performed after DAPI addition and mounting on a Leica THUNDER Imager and a Leica SP8 confocal microscope (Leica Microsystems). All images stored at the OMERO platform,

maximum intensity projections were produced using the OMERO images, labeling was performed with Inkscape®.

Phagocytosis assay with GFP-stained particles. Wt and *Tnfsf14*$^{-/-}$ TR-AMs were compared regarding their phagocytosis capacity on day 7 pi, using the pHrodo^{TM} Green *Escherichia coli* BioParticles^{TM} phagocytosis kit for flow cytometry (cat. P35366, Invitrogen) as per the manufacturer´s instructions. BALF cells were isolated from IAV-infected mice as previously described and resuspended in 1mL FACS buffer (PBS, 7.4% EDTA, 0.5% FCS, pH 7,2, 0,01% NaAz), which was then divided in four samples. Samples were centrifuged at 1.5g for 5min and resuspended in 0.1mL FACS buffer. pHrodo BioParticles™ were resuspended in 2 mL of buffered saline solution to generate a 1 mg/mL stock suspension. Two of the samples received 0.01mL particle suspension and the other two saline solution. One sample containing particles and one containing empty solution were incubated at 4°C or 37°C for 30min. After that, all samples were placed on ice for 1min to stop phagocytosis. Cells were washed and resuspended in leukocyte surface staining for 15min at 4°C. Phagocytosis capacity was evaluated via flow cytometry as a combination of % FITC-positive TR-AMs and quantification of FITC signal normalized to control samples. Total FITC signal was calculated as follows: $(MFI_{particles}-MFI_{empty})_{37°C}-(MFI_{particles}-MFI_{empty})_{4°C}$.

Alveolar epithelial cell (AEC) isolation and treatment. To test any apoptosis-inducing effect of TNFSF14 after IAV infection on AECs, lung homogenates from wt mice were obtained by instillation of dispase (cat. 354235, Corning) through the trachea into HBSS (cat. 14175-053, Gibco) perfused lungs, followed by incubation in dispase for 40min at RT. After removal of the trachea and proximal bronchial tree, lungs were homogenized (GentleMACS, MACS Miltenyi Biotechnologies) in DMEM/2.5% HEPES with 0.01% DNase (cat. 18535.02, Serva) and filtered through 100 and 40 µm nylon filters. Cell suspensions were incubated with biotinylated rat anti-mouse CD45 (clone

30-F11, cat. 553078, BD Biosciences), rat anti-mouse CD16/32 (clone 2.4G2, BD, cat. 553143, BD Biosciences), and rat anti-mouse CD31 (clone MEC13.3, cat. 553371, BD Biosciences) antibodies for 30min at 37 °C, followed by incubation with biotin-binding magnetic beads and magnetic separation to deplete leukocytes and endothelial cells prior to further culture. AEC suspensions with a purity ≥90% as determined by flow cytometry were seeded at a density of 300,000 cells in 4 μm pore size transwells (cat. 3470, Corning® Costar®) to generate polarized monolayers, and cultured in DMEM enriched with HEPES, L-glutamine, FCS, and penicillin/streptomycin. Five days later, cells were infected with IAV at an MOI 0.1, and treated with rTNFSF14 (Cat. 1794-LT, R&D Systems) at a concentration of 500ng/ml for 24h. TNFSF14-containing medium was added either directly on the transwell where the AECs were seeded, with the well underneath containing TNFSF14-free medium, or vice versa. AEC survival was analyzed via flow cytometry (% annexin V^-/7-AAD^- cells).

ELISA. Soluble TNFSF14 was measured in murine and human BALF samples using commercially available ELISA kits (murine TNFSF14: R&D Systems, cat. DY1794-05, detection limit 250pg/mL, human TNFSF14: R&D Systems, cat. DLIT00, detection limit 16.5pg/mL), according to the manufacturer's instructions.

SDS-PAGE and native PAGE. To determine if soluble TNFSF14 adopts a (homo)trimeric, active, structure in the BALF of IAV-infected mice, samples from non-infected mice and IAV-infected mice on day 3 pi (when a significant increase in BALF TNFSF14 could first be observed, Figure 3I) were subjected to protein denaturation and reduction or prepared in native sample buffer (cat. 161-0738, Bio-Rad) with additional Coomassie® Brilliant Blue G 250 (0.1%; w/v, cat. 27815-25G-F, Sigma-Aldrich) to the outer surface of protein complexes leading to a negatively charged protein-dye complex. Recombinant mouse TNFSF14 (rTNFSF14, cat. 1794-LT-025 R&D Systems) was used as a positive control. BALF samples were concentrated using

Amicon® Ultra 0.5 mL Centrifugal Filters (cat. UFC5003BK, Merck), prior to sample preparation for electrophoresis. SDS-PAGE was performed for denaturated, reduced samples as previously published (1). Native protein samples were used for native PAGE according to the protocol provided by the manufacturer (Invitrogen, Thermofisher). All samples were transferred to PVDF membranes (Trans-Blot Turbo Transfer Pack, cat. 704156, Bio-Rad) and immunodetection was conducted using an anti-TNFSF14 antibody (rabbit anti-mouse, cat. ab203578, 1:500, abcam) and a goat anti-rabbit secondary antibody (cat. W4011, 1:5000, Promega). Images were acquired using ChemiDoc Imaging Systems (Bio-Rad).

Quantitative PCR. RNA isolation was performed with the RNeasy kit (Qiagen) and cDNA was synthetized according to the manufacturer´s instructions. Quantitative PCR (qPCR) was then performed with SYBR green I (InvitrogenTM) in the StepOnePlusTM Real-Time PCR System and in the QuantStudio™ 3 Real-Time PCR System (Applied Biosystems^{TM}). *Gapdh* and *RPS18* expression served as normalization controls. Data are presented as ΔCt (Ct reference – Ct gene of interest). The following primers were used:

a) murine: *Gapdh* (forward primer, 5´-AGGTCGGTGTGAACGGATTTG-3´; reverse primer, 5´-GGGGTCGTTGATGGCAACA-3´), *Tnfsf14* (forward primer, 5´-ACTGCATCAACGTCTTGGAGA-3´; reverse primer, 5´-TGGCTCCTGTAAGATGTGCTG-3´), *Tnfrsf14* (forward primer, 5´-CAGGCCCCTACAGACAACAC-3´; reverse primer, 5´-ACTCGTCTCCCACAAGGAACT-3´), *Ltbr* (forward primer, 5´-CCCCTTATCGCATAGAAAACCAG-3´; reverse primer, 5´-TGCATACCGCAAAGACAAACT-3´)

b) human: *TNFSF14* (forward primer, 5´-GGTCTCTTGCTGTTGCTGATGG-3´; reverse primer, 5´-TTGACCTCGTGAGACCTTCGCT-3´), *RPS18* (forward primer, 5´-

GCAGAATCCACGCCAGTACAAG-3´; reverse primer 5´-GCTTGTTGTCCAGACCATTGGC-3´).

RT^2 Profiler PCR Arrays data analysis. For gene expression analysis in the different comparison groups, a threshold of Ct 30 was defined to exclude undetected expression (Ct >30). Fold-Change (2^(-ΔΔCt)) is the normalized gene expression (2^(-ΔCt)) in the IAV-infected samples divided by the normalized gene expression (2^(-ΔCt)) in mock-infected alveolar macrophages. *Gapdh* was used as the housekeeping gene for normalization of gene expression within each sample, log2 fold change values are presented for each gene. P-values were calculated based on 2-tailed Student's t-test of each gene´s log2 fold change values in the compared groups. A star has been added next to log2 fold change values with a p-value< 0.05 to indicate significance. Ct values for each gene and sample, average Ct values, standard deviation in average Ct and ΔCt values, fold change of genes in infected over mock-infected samples, and p-values, can be found in the spreadsheet files ´Cell death arrays wt data´, ´TNF signaling arrays wt data´, and ´Cell death arrays *Tnfsf14* ko data´ of the Supplemental Material accompanying the main manuscript as well as in the Supporting data values file. Whenever Ct values either in the mock or in the infected group were higher than our cut-off (Ct 30), a specific letter A-B was added next to the fold change value as presented in the spreadsheets, aiming at better illustrating these variations. A: The gene's average threshold cycle was relatively high (>30) in either the mock or the infected sample and reasonably low in the other sample (<30), suggesting that the actual fold-change value is at least as large as the calculated and reported fold-change result. B: The gene's average threshold cycle was relatively high (>30), meaning that its relative expression level was low, in both mock and infected samples, and the p-value for the fold-change was either unavailable or relatively high (p >0.05).

Neutrophil isolation from human BALF samples. Neutrophil isolation from the BALF of patients with IAV ARDS and patients who underwent bronchoscopy for diagnostic purposes was performed with the MACSxpress® Whole Blood Neutrophil Isolation Kit (cat. 130-104-434, Miltenyi), according to the manufacturer´s instructions.

References

1. Hadzic S, et al. Fibroblast growth factor 10 reverses cigarette smoke- and elastase-induced emphysema and pulmonary hypertension in mice. Eur Respir J. 2023;62(5).
